# A multigene mutation classification of 468 colorectal cancers reveals a prognostic role for *APC*

**DOI:** 10.1038/ncomms11743

**Published:** 2016-06-15

**Authors:** Michael J. Schell, Mingli Yang, Jamie K. Teer, Fang Yin Lo, Anup Madan, Domenico Coppola, Alvaro N. A. Monteiro, Michael V. Nebozhyn, Binglin Yue, Andrey Loboda, Gabriel A. Bien-Willner, Danielle M. Greenawalt, Timothy J. Yeatman

**Affiliations:** 1 Department of Biostatistics and Bioinformatics, Moffitt Cancer Center and Research Institute, 12902 Magnolia Drive, Tampa, Florida 33612, USA; 2 Gibbs Cancer Center and Research Institute, 380 Serpentine Drive, Spartanburg, South Carolina 29303, USA; 3 Genomic Services, LabCorp Clinical Trials, 401 Terry Avenue North, Suite 200, Seattle, Washington 98109, USA; 4 Department of Anatomic Pathology, Moffitt Cancer Center and Research Institute, 12902 Magnolia Drive, Tampa, Florida 33612, USA; 5 Department of Epidemiology, Moffitt Cancer Center and Research Institute, 12902 Magnolia Drive, Tampa, Florida 33612, USA; 6 Genetics and Pharmacogenomics, Merck, Sharp and Dohme, PO Box 4, 770 Sumneytown Pike, Building 53, West Point, Pennsylvania 19486, USA; 7 Molecular Health, 2700 Technology Forest Boulevard, The Woodlands, Texas 77381, USA

## Abstract

Colorectal cancer (CRC) is a highly heterogeneous disease, for which prognosis has been relegated to clinicopathologic staging for decades. There is a need to stratify subpopulations of CRC on a molecular basis to better predict outcome and assign therapies. Here we report targeted exome-sequencing of 1,321 cancer-related genes on 468 tumour specimens, which identified a subset of 17 genes that best classify CRC, with *APC* playing a central role in predicting overall survival. *APC* may assume 0, 1 or 2 truncating mutations, each with a striking differential impact on survival. Tumours lacking any *APC* mutation carry a worse prognosis than single *APC* mutation tumours; however, two *APC* mutation tumours with mutant *KRAS* and *TP53* confer the poorest survival among all the subgroups examined. Our study demonstrates a prognostic role for *APC* and suggests that sequencing of *APC* may have clinical utility in the routine staging and potential therapeutic assignment for CRC.

## Abstract (web summary)

*APC* is a well-known tumour suppressor that is frequently inactivated in colorectal cancer. Here, the authors sequence more than 1000 cancer genes in 468 colorectal cancers and show that mutation signatures can be used to classify the tumours and that multiple mutations in *APC* are associated with a poor prognosis.

## Graphical abstract

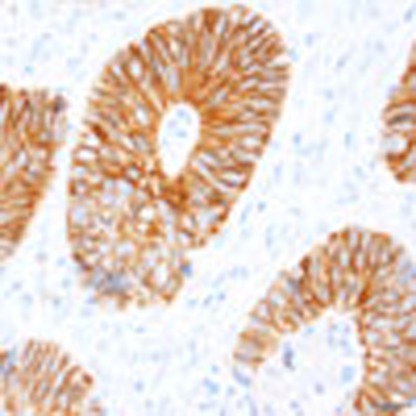

Colorectal cancer (CRC) is a highly heterogeneous disease with diverse genetic and clinical features that influence therapeutic outcomes^1^,^2^. Clinicopathologic staging has been the mainstay of CRC prognosis since Cuthbert Dukes first described it in 1932 (ref. 3). With the emergence of nextgen sequencing technologies, it is now possible to extensively and rapidly evaluate a large number of genes and samples to inform its diagnosis, prognosis and potential response to therapy. Thus, a robust and detailed molecular disease classification—beyond the few currently measured molecular parameters (MSI, *KRAS*, *NRAS* and *BRAF*)—is urgently needed to better understand and treat this disease^4^,^5^,^6^. One major challenge is to identify the multivariable molecular factors that optimally predict distant metastasis and outcome. Recently, The Cancer Genome Atlas (TCGA) project has profiled and characterized the landscape of CRC mutations^7^. While the TCGA project robustly characterized individual mutational events in 224 samples, their interaction and impact on metastasis and survival was not fully evaluated.

*Adenomatous polyposis coli (APC)* has long been considered the ‘gatekeeper' gene for the vast majority of CRCs, and, for this reason, its discriminating functionality in predicting prognosis may have been overlooked in the past. *APC* is a tumour-suppressor gene whose protein product functions as an antagonist of the WNT signalling pathway by binding and regulating the β-catenin protein^2^. *APC* may be also involved in other cellular processes including cell migration and adhesion, transcriptional activation, apoptosis and DNA repair^8^,^9^. *APC* inactivation is thought to be an early event in the development of CRC, and may play a pivotal role in the initiation of the adenoma–carcinoma pathway^2^,^10^. In recent years, various studies on molecular classification of CRC have been reported^5^,^6^,^7^,^11^,^12^,^13^,^14^. Although *APC* is the most frequently mutated, known driver gene in CRC^2^, it has not generally been included as a factor in clinical prognostic classification and is not generally included in standard CRC-sequencing panels.

In this study, we performed TGS for 1,321 cancer-related genes and microsatellite instability (MSI) analysis on an independent set of 468 clinically characterized, sporadic, colorectal tumours. We present a multigene interactive classification analysis for colorectal carcinoma, which groups individual mutations based on their natural associations with each other. Clinical covariates, including metastasis and survival, are also provided to help further define the prognostic potential of the classification system, with *APC* mutations identified to play a central role. Subsequent clinical associations result in a prognostic, five-class classification that demonstrates a previously unknown, prognostic role of the *APC* mutation status in association with *APC* ‘partnering mutations'.

## Results

### Identification of 17 significantly mutated genes

A targeted gene sequencing (TGS) analysis was performed on 468 colorectal tumour samples across 1,321 genes known or highly suspected to be associated with human cancer (Supplementary Table 1). To evaluate the MSI status, the Bethesda panel analysis^15^ was performed on all 468 CRC cases, yielding 61 (13%) MSI-H samples (called MSI henceforth). We found that 59/61 (97%) of MSI tumours had both *TGFBR2* and *ACVR2A* insertion/deletion (indel) mutations in homopolymer regions of the targeted genes. Meanwhile, only one of three tumours with *TGFBR2*, but not *ACVR2A*, and one of six tumours with the reverse profile were MSI. Thus, the co-occurrence of mutations of these two genes nearly perfectly characterizes the MSI status. In support of this notion, *ACVR2A* and *TGFBR2* were also reported by TCGA as the top two representative genes in hypermutated tumours, 77% of which were MSI^7^.

**Figure 1:**
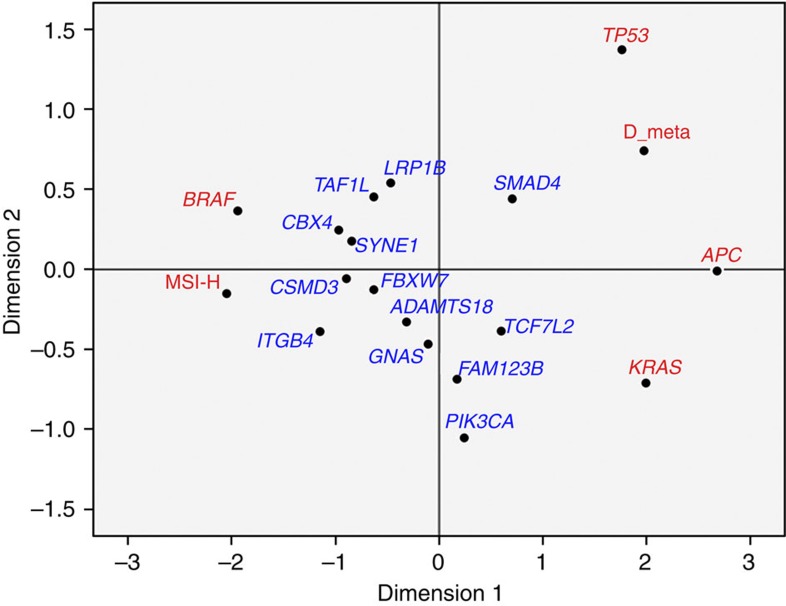
Multidimensional scaling plot for the correlation of highly mutated genes. This plot (Euclidean distance model) graphically depicts the correlation of the 17 genes, with elevated non-silent mutation rates presented in Table 2 along with MSI-H status and presence of distant metastasis (D-meta). The most significant correlated genes *BRAF, APC, KRAS, TP53* and MSI-H and D-meta are highlighted in red. *BRAF* is very close to (having strong positive correlation with) MSI-H but is anticorrelated with (far apart from) distant metastasis. Conversely, the partnering mutations (*APC, KRAS* and *TP53*) are comparatively close to each other and are with distant metastasis. Notably*, ITGB4*, *CSMD3* and *CBX4* are much closer to MSI-H than D-meta on the plot. *FAM123B*, also called *AMER1*, is a chromosome X-linked mutation, and 38/51 patients with mutations are female.

Among the 1,321 targeted genes, a robust regression analysis versus amino-acid length identified 22 genes with elevated non-silent mutation rates at a *z*-score of ⩾3 (Table 1 and Supplementary Data 1), either overall, or for MSI or MSS (microsatellite stable) tumours. Our method has independently confirmed the significantly mutated genes identified by the TCGA group in non-hypermutated CRC tumours. We have re-identified all genes seen at ⩾7% frequency in that data set. Five of our identified genes, however, had statistically significant (*P*<0.0001, Fisher Exact) mutation rates that were much lower in TCGA samples (Supplementary Table 2). As the TCGA mutations were determined using paired tumour/normal data, which is the more precise method for determining somatic mutations, we restricted our attention to the remaining 17 genes that had similar mutation frequencies in the TCGA data set (*KRAS, TP53, APC, SMAD4, FBXW7, BRAF, TCF7L2, PIK3CA, GNAS, CBX4, ADAMTS18, TAF1L, FAM123B, CSMD3, ITGB4, LRP1B* and *SYNE1*, given in decreasing order of mutation rate). We next calculated ranking of correlation of these genes with the four key driver genes identified in our study (*BRAF, APC, KRAS* and *TP53*), MSI status, distant metastasis status, EMT (epithelial–mesenchymal transition) and RAS/MAPK gene expression signature scores^16^,^17^ (Table 2). The genes are sorted based on increasing MSI correlation percentile, which is strongly inversely correlated with distant metastasis (Spearman *r*=−0.84). *APC*, *TP53* and *KRAS* (and *SMAD4* to a lesser extent) are shown to be among the most strongly *negatively* associated with MSI but positively associated with metastasis (Table 2 and Fig. 1). When limited to only MSS patients (*N*=407), mutations in these three genes are strongly associated with metastasis (*P*=0.0051, logistic regression *χ*^2^), with *KRAS* having the strongest individual association (*P*=0.0010, *χ*^2^ test). Notably, our large cohort confirms the inverse relationship of *APC* mutations with MSI previously reported by others^7^,^18^,^19^,^20^,^21^. We selected these frequently mutated *APC*, *TP53* and *KRAS* genes to characterize this MSS group. *TCF7L2* and *FAM123B* are WNT signalling genes and have much weaker tendencies, but tend to associate with the genes listed above. By contrast, *BRAF, ITGB4, CBX4, CSMD3, SYNE1, FBXW7* and *TAF1L* are strongly correlated with MSI and non-metastatic leaning genes. The remaining four genes, including *PIK3CA*, lie in between these contrasting groups (Table 2 and Fig. 1).

### Four driver genes had pairwise and significant correlations

As the single gene mutation status does not capture the full complexity of a tumour, we classified our samples via mutation combinations in those genes determined to be commonly mutated, but varying across samples. Among the 17 genes, the most striking negative Spearman correlations were *BRAF* and *KRAS*, and *BRAF* and *APC* (both with *P*<0.0001), while the highest positive association (Spearman *r*=0.26, *P*<0.0001) was between *APC* and *KRAS* (Table 2). The only remaining positively associated gene with *APC* with Fisher *P*<0.01 was *TP53.* Given the strength of these associations and their high mutation frequencies, these four genes were used to comprise the basis of a 10-group classification as shown in Supplementary Fig. 1A. Notably, our data and TCGA data have similar distribution patterns across multigene mutation groups in the 10-group analysis. In our cohort, the distribution patterns of MSI and MSS tumours differ remarkably (Supplementary Fig. 1B), supporting the notion that these CRC types undergo tumorigenesis through very different genetic mechanisms, and, accordingly, have different clinical outcomes^22^,^23^.

### *BRAF* and *APC/KRAS/TP53* partnering defines MSI and metastasis

Demographic features of the 10 groups are shown in Supplementary Table 3. While the 10-group analysis lays out the mutational patterns in detail, some trending becomes clearer by counting the number of mutated partnering genes (*APC*, *KRAS* and *TP53*). Eighty-four per cent of *BRAF*-only tumours are MSI compared with only 50% when a *TP53* mutation is also present. Of these 45 *BRAF*-grouped tumours (B or BP), only 9 (20%) were metastatic. In contrast, among the 199 tumours with both *APC* and *TP53* mutations, only 1% (2) are MSI, while 43% have metastatic disease.

The observed counts of the four *APC*-mutated groups (A, AK, AP and AKP) show that *APC* usually co-occurs with either *KRAS* or *TP53* mutations, or both (Supplementary Table 3); of 312 such tumours, only 43 (14%) are in Group A (*APC* only), suggesting that *APC* mutations need to partner with one or more additional driver mutations to advance to CRCs. Interestingly, the triple-mutant AKP (*APC/KRAS/TP53*) group had the highest metastatic rate, with 22% developing metastatic disease from a stage 1–3 primary, suggesting a possible evolution from either AK or AP mutants by adding the third partner mutation. Primary site location might also be a deciding factor here (Supplementary Table 3). Notably, in the caecum and ascending right colon, AKPs outnumber APs and AKs, whereas in the other locations the number of APs always exceeded that of AKPs, especially in the rectosigmoid and sigmoid locations.

While *APC* mutations were highly associated with both *KRAS* (Fisher *P*<0.0001) and *TP53* (Fisher *P*=0.0042) mutations, the latter are marginally negatively associated with each other (Fisher *P*=0.066), as has been reported by other groups^4^,^20^. This negative association is confined to the *APC*-mutated group (Fisher *P*=0.0005; Supplementary Table 4). These observations (*APC* is less frequently mutated alone, more commonly mutated with *KRAS*, *TP53* or both) suggest that the *APC* mutation occurs early in carcinogenesis, a result corroborated by the high frequency of *APC* mutations in adenomas^2^,^10^. However, it is noteworthy that, although *APC*→*KRAS*→*TP53* is a widely perceived sequential pathway of the key driver mutation events commonly occurring in CRC development^1^,^10^,^24^, our data provide further clues in the development of CRC involving multiple pathways, as 156 tumours lack an *APC* mutation.

### Analysis of *APC* mutational status

We further analysed *APC* mutational status in association with MSI, allelic loss and other non-*APC* WNT pathway mutations (Table 3 and Supplementary Table 5). Among *APC*-mutated tumours, 38% (118/312) displayed two or more truncating mutations. Only five tumours had more than two *APC* mutations. These data provide a compelling case for the significance of the second mutation, supporting the bi-allelic nature of *APC* because of its deviation from a Poisson distribution (*χ*^2^ goodness of fit *P*=2.6 × 10^−11^). We then divided the *APC* gene into four zones based on their truncating mutation rates (Table 3). Zones 1 (codons 1–218) and 4 (codons 1,588–2,843) had very few such mutations (*N*=3 for zone 1; *N*=2 for zone 4, both in one *POLE* tumour that also had two other *APC*-truncating mutations). Zone 3 (codons 1,263–1,587) had a high mutation rate (259 mutations over 325 amino acids), and this zone has been deemed the ‘mutation cluster region'^25^ because of the high rate of germline mutations in familial adenomatous polyposis (FAP)-associated colon cancer. Zone 2 (codons 219–1,262) had a moderately high rate of *APC* mutations (*N*=181 over 1,044 amino acids). With striking statistical significance (*χ*^2^ *P*<1 × 10^−15^), tumours with two *APC* mutations had one mutation each from zones 1–3. This is in accord with the double mutation profile established by others^26^,^27^, more specifically for FAP colon cancers. Zone 3 mutations would yield 1–3 (out of 14) functional 20-amino-acid β-catenin-binding sites per molecule and zone 1–2 mutations would remove all β-catenin-binding sites. This further supports the notion that ‘just right' WNT/beta-catenin signalling may be necessary for advanced progression of CRC^21^,^26^,^27^. Interestingly, most rare exceptions to this *APC* double-hit pattern were either in variant isoforms or were hypermutated *POLE* tumours^7^ (Table 3).

On the other hand, it was also observed that almost 30% of tumours harboured only one *APC* mutation without inferred allelic loss, most of which were MSS tumours (Table 3), with a substantial number also lacking any other WNT pathway mutation from our examined list. Analysis of the published TCGA data set (including somatic mutations and copy-number (CN) alterations) shows a similar result: 35% (74/209) of tumours harbour a one-hit mutation in *APC* without CN loss (Supplementary Table 6), which actually exceeds the number of samples with two APC hits (mutations and/or CN loss): 34% (71/209). Moreover, an independent mutation and a loss of heterozygosity (LOH) analysis of 62 additional CRC tumours were carried out on the selected drivers. While *APC*, *TP53*, *KRAS/NRAS* and *BRAF* showed similar mutation patterns, the rate of *APC* allelic loss appeared to be low and was observed only in tumours with *APC* 0- and 1-truncating mutation (Supplementary Data 2).

### Immunohistochemical analysis of β-catenin nuclear staining

**Figure 2:**
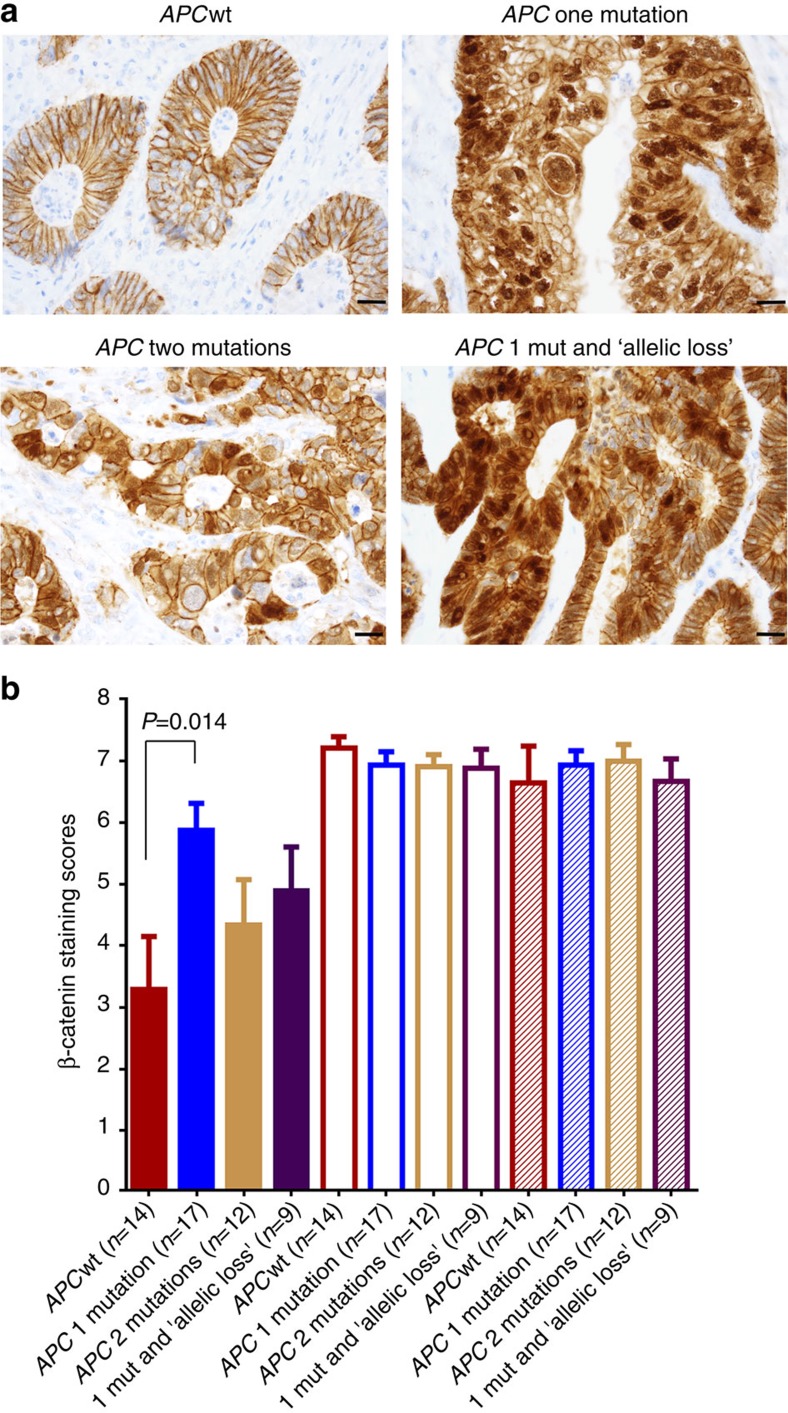
Immunohistochemical staining of β-catenin in 52 colorectal cancers. (**a**) Representative images for *APC* groups (*APC*wt, one mutation, two mutations and one mutation plus inferred allelic loss ‘LOH'). Scale bar (bottom right), 20 μm. (**b**) Allred scores for nuclear (solid bars), cyto (unfilled bars) and membranous (hatched bars) staining of β-catenin in the *APC* groups: *APC*wt (*n*=14), one mutation (*n*=17), two mutations (*n*=12) and one mutation plus inferred allelic loss ‘LOH' (*n*=9). Error bar represents s.e.m. *P*=0.014 is for two-tailed, unequal variance Welch *t*-test. See detailed score information for individual tumours in Supplementary Table 7.

To determine whether the WNT/β-catenin pathway might be activated in tumours with one *APC*-truncating mutation, we performed an immunohistochemical (IHC) analysis of 52 FFPE (formalin-fixed, paraffin-embedded) tumours (selected from 468 CRCs) with a β-catenin antibody. Results show that β-catenin nuclear staining in *APC* single-mutation tumours was significantly stronger than *APC*wt tumours (Welch *t*-test *P*=0.014, Fig. 2a,b), indicating *increased* β-catenin activities in these *APC*-mutated tumours. For example, a ‘zero' nuclear staining score was seen in six *APC*wt tumours (out of 14 cases), but in only one single-mutation tumour (out of 17 cases; Supplementary Table 7). Significantly increased nuclear staining was not demonstrated in tumours with either *APC* multiple mutations (*n*=12, Welch *t*-test *P*=0.37) or single mutation plus inferred allelic loss (*n*=9, Welch *t*-test *P*=0.17), possibly because of small sample sizes (Fig. 2b).

### Activation of the WNT/β-catenin pathway in *APC* subgroups

We further investigated WNT/β-catenin activation in 458 CRCs (10 samples without suitable microarray data were excluded), which were divided into four *APC* groups: (1) *APC*wt (*n*=151), (2) 1 truncating mutation without inferred allelic loss (*n*=124), (3) 2+ truncating mutations (*n*=124) and (4) 1 truncating mutation plus inferred allelic loss (*n*=59). A complete set of 64 β-catenin (upregulated)-targeted genes identified in CRC cell lines by Herbst *et al*.^28^ was used as a measure of WNT pathway activation. Results show that the WNT targets were significantly activated in *APC* single-mutation tumours without allelic loss (Welch *t*-test *P*<0.0001), and in tumours with *APC* 2+ mutations (Welch *t*-test *P*=0.0004) or with single mutation plus allelic loss (Welch *t*-test *P*=0.011; Supplementary Fig. 2A). In addition, no statistically significant difference was seen between zone 1–2 and 3–4 mutations in tumours containing *APC*-one-truncating mutation either without or with inferred allelic loss (Supplementary Fig. 2B). These data support the notion that the WNT/β-catenin pathway was significantly activated in the *APC*-mutated tumours, which appeared to be zone-independent.

### *APC* expression and methylation and other WNT pathway genes

We also investigated a possible role of methylation of the *APC* gene as a ‘second hit' using the TCGA CRCs^7^, of which 209 samples had mutation, CNA and RNAseq expression data. Here we identified ‘hits' in *APC* by counting the number of truncating mutations and CN deletions in each sample. We then compared the number of hits to mRNA expression (RNASeq) of the genes of interest. We first examined *APC* mRNA expression and observed a general trend of decreasing *APC* expression, with increasing number of *APC* hits (Supplementary Fig. 3). Differences between 0, 1 and 0, 2 *APC* hits were significant, but only the difference between 0 and 2 hits was significant after Bonferroni–Holm correction. This difference could be related to mRNA instability induced by truncation. We then examined *APC* methylation profiles and the relationship to *APC* expression in each *APC* mutation group. Promoter-specific probe cg15020645 (Supplementary Fig. 4 and Methods) showed the bimodal pattern often observed in promoter methylation; however, no significant differences were observed between *APC*-hit groups using the Wilcoxon rank-sum test (Supplementary Fig. 5A). Although the methylation pattern was different in probe cg01240931 (Methods), no significant differences were observed between *APC*-hit groups (Supplementary Fig. 5B). Indeed, neither probe showed any difference between one and two *APC* hits, strongly suggesting that *APC* promoter methylation is not a second hit in *APC* one-hit *APC* samples. In addition, we observed no significant correlation between *APC* promoter methylation and *APC* gene expression in either *APC* one-hit or all samples (Supplementary Fig. 6).

In addition to *APC* methylation, we also investigated a possible role of non-*APC*-mutated WNT pathway gene mutations as a ‘second hit' in one-hit- *APC* mutation tumours using the TCGA CRCs that were available with whole-exome-sequencing data. Of 67 WNT pathway genes examined, 63 had mutations and were analysed (Methods). We found no significant correlation between the number of mutated non-*APC* WNT pathway genes and the expression of the 64 β-catenin-targeted genes (Pearson *r*=−0.03, *P*=0.67, Supplementary Fig. 7), suggesting that these non-*APC* WNT gene mutations are not responsible for WNT pathway activation. We then assessed the frequency of these non-*APC* WNT gene mutations versus the number of *APC* hits, and found that more mutations were associated with zero-hit *APC* (Supplementary Fig. 8). In addition, no differences were seen between one- and two-hit *APC* groups. Taken together, mutations in WNT pathway genes other than *APC* did not appear to be a ‘second hit' in the majority of one-hit *APC* samples.

### Prognostic role of *APC* and the five-class classification

**Figure 3:**
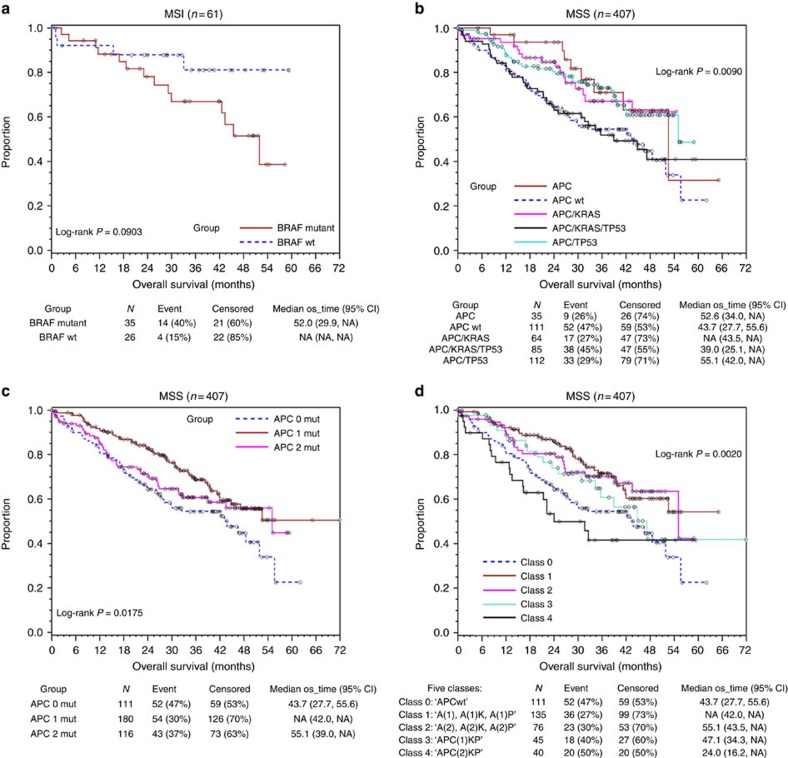
Kaplan–Meier survival analysis. (**a**) MSI tumours by *BRAF* mutation, (**b**) MSS tumours by *APC* groups (*APC*wt, *APC*, *APC*/*KRAS*, *APC*/*TP53*, *APC*/*KRAS*/*TP53*), (**c**) MSS tumours by the number of truncating mutations (0, 1, 2) in the *APC* gene, (**d**) MSS tumours by both *APC* groups and the number of *APC* mutations. A−*APC*, K−*KRAS*, P*−TP53*. Five classes: Class 0: *APC* wild type; Class 1: *APC(1), APC(1)*/*KRAS*, *APC(1)*/*TP53*; Class 2: *APC(2), APC(2)*/*KRAS*, *APC(2)*/*TP53*; Class 3: *APC(1)/KRAS*/*TP53*; Class 4: *APC(2)/KRAS*/*TP53.* A(1) or *APC* (1) represents one *APC* mutation and A(2) or *APC* (2) represents two *APC* mutations.

For prognostic analysis of 468 CRCs, we first divided patients by their MSI status for clinical evaluation because of the strong negative association between the MSI status and distant metastasis, and their sharp contrast in mutation counts (median=169 for MSI and 33 for MSS tumours). Among MSI tumours, normally considered to carry a good prognosis overall^22^,^23^, Kaplan–Meier analysis shows that *BRAF* (*V600E*)-mutated tumours have marginally significantly poorer overall survival (log-rank *P*=0.090; Fig. 3a).

In MSS tumours, overall survival was roughly equivalent among the *APC*, *APC*/*KRAS* and *APC*/*TP53* groups, but lower in the *APC*/*KRAS*/*TP53* (AKP) group, which was nearly equivalent to that seen among *APC*wt patients (log-rank *P*=0.0090; Fig. 3b). Notably, our association analyses with two signature scores indicate that, while the AKP triple mutants had higher RAS/MAPK activity^16^, *APC*wt tumours were associated with a higher EMT score^17^,^29^ (Supplementary Fig. 9), suggesting that AKP and *APC*wt might undergo distinct mechanisms of tumorigenesis and thereby differ in drug responsiveness. In support of this notion, our previous study showed that EMT was inversely associated with RAS activity^17^. Moreover, *APC* two-mutation- and zero-mutation tumours showed worse survival compared with *APC* one mutation tumours (log-rank *P*=0.018; Fig. 3c). Since both AKP triply mutated and other *APC*-mutated tumours had either one or two *APC* mutations, we further re-grouped these MSS tumours into five classes: Class 0 (*n*=111): ‘*APC*wt'; Class 1 (*n*=135): ‘*APC*(1), *APC*(1)*/KRAS*, *APC*(1)/*TP53*'; Class 2 (*n*=76): ‘*APC*(2), *APC*(2)/*KRAS*, *APC*(2)/*TP53*'; Class 3 (*n*=45): ‘*APC*(1)/*KRAS*/*TP53' (APC(1)*KP for short); and Class 4 (*n*=40): ‘*APC*(2)/*KRAS*/*TP53'* (*APC(2)KP* for short). Here *APC*(1) represents one *APC* mutation and *APC*(2) represents two *APC* mutations. This five-class classification incorporates both *APC* mutation count and partnering mutation count, and results in more significantly resolved survival analysis (log-rank *P*=0.0020; Fig. 3d). Compared with Class 1 (reference) tumours, while *APC*wt (Class 0) tumours still carried substantially worse survival (hazard ratio (HR)=1.94, *χ*^2^ *P*=0.0023), the AKP triply mutated tumours with two *APC* mutations (Class 4) had the worst survival (HR=2.48, *χ*^2^ *P*=0.0011). However, the AKP tumours with one *APC* mutation (Class 3) and other *APC* two-mutation (Class 2) tumours were no longer significantly associated with worse survival (HR=1.48, *χ*^2^ *P*=0.17 and HR=1.11, *χ*^2^ *P*=0.67, respectively) in this refined model, suggesting that either AKP or two *APC* mutations alone were separately insufficient for prediction of worse survival.

**Figure 4:**
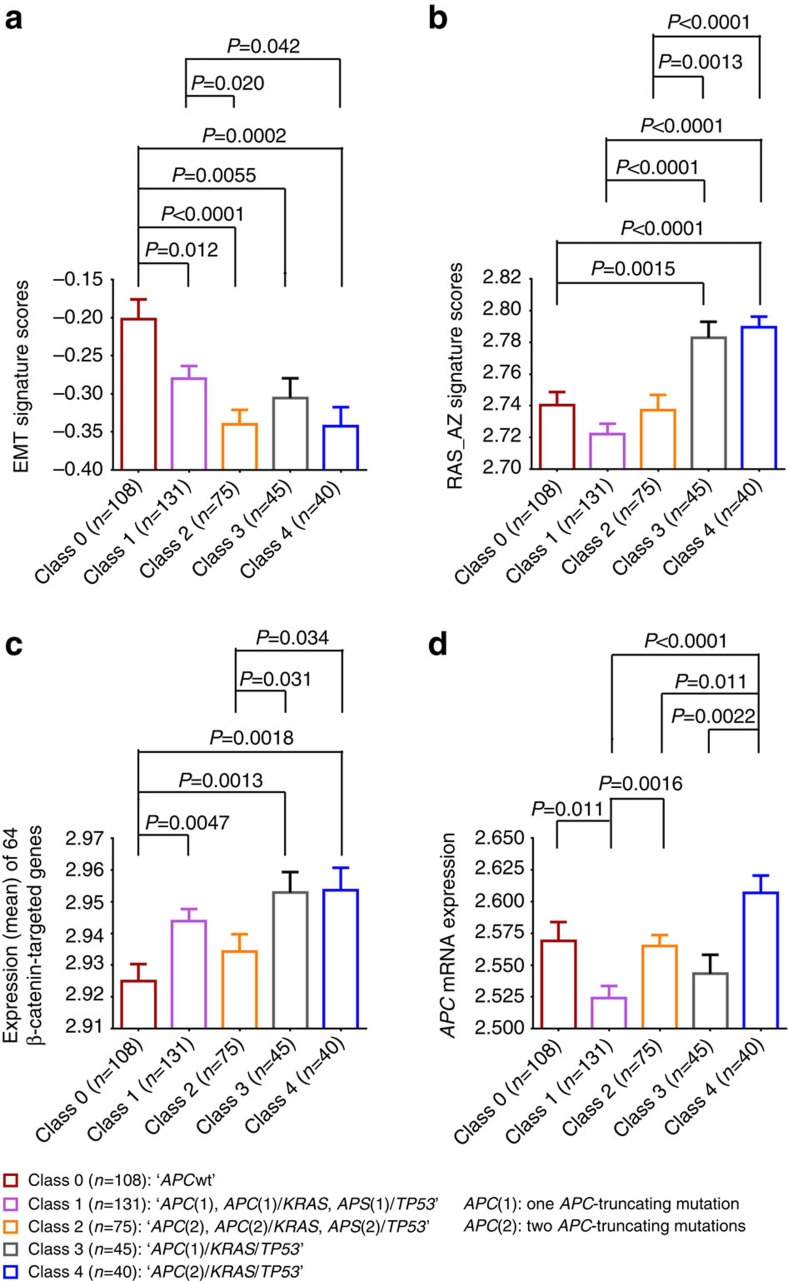
Expression (log2) comparison of MSS tumours in five classes (*n*=399). (**a**) EMT signature scores^17^,^29^, (**b**) RAS_AZ signature scores^16^, (**c**) the 64 β-catenin-targeted genes^28^ and (**d**) *APC* mRNA expression (mean of eight *APC* probes). Five classes: Class 0: *APC* wild type; Class 1: *APC(1), APC(1)*/*KRAS*, *APC(1)*/*TP53*; Class 2: *APC(2), APC(2)*/*KRAS*, *APC(2)*/*TP53*; Class 3: *APC(1)/KRAS*/*TP53*; Class 4: *APC(2)/KRAS*/*TP53. APC* (1) represents one *APC* mutation and *APC* (2) represents two *APC* mutations. Error bars represent s.e.m. The two-tailed, unequal variance Welch *t*-test was used to assess the statistical significance of comparison. Unadjusted *P*-values of <0.05 are shown. Notably, eight samples without suitable microarray data were excluded from 407 MSS CRCs. Class 0 tumours were significantly more ‘mesenchymal-like' than all four other classes (**a**), while AKP-related Classes 3 and 4 had stronger RAS activation (**b**). Compared with Class 0 tumours, WNT activation, as measured by the 64 WNT target genes, was observed in *APC*-mutated Class 1 (*P*=0.0047), Class 3 (*P*=0.0013) and Class 4 (*P*=0.0018; **c**).

The five-class classification for all patients (*n*=468) is given in Table 4. To biologically analyse this differential behaviour, expression comparison between the five classes was also performed on 399 MSS tumours (from 458 CRCs with complete data) using the EMT^17^ and RAS/MAPK^16^ signatures as well as the 64 β-catenin-targeted genes^28^ (Fig. 4a–c). Notably, despite their different impacts on survival, no significant differences were seen between Class 3 and Class 4 in the EMT scores, RAS scores or the expression of the WNT/β-catenin targets (Fig. 4a–c). We also assessed *APC* mRNA expression in the five classes and found that Class 4, which had the worst survival, also had the highest mRNA expression, which was significantly above the less lethal classes (Class 1, Welch *t*-test *P*<0.0001, Class 2, *P*=0.011 and Class 3, *P*=0.0022) but was also marginally significantly higher than the Class 0, which also had poor survival (Welch *t*-test *P*=0.062; Fig. 4d). Notably, *APC* mRNA expression of the tumours that had two *APC* mutations was significantly higher than that of their respective one *APC* mutation counterparts (that is, Class 2 versus Class 1 and Class 4 versus Class 3); however, Class 4 tumours also had stronger RAS pathway activation and higher expression of WNT targets than Class 2 tumours (Fig. 4b,c).

### Cox model predicators of survival and CMS classification

Cox modelling was used to determine the effect of the four key genes on overall survival among MSS tumours, with only an adjustment for older age (Table 5; Model 1). An *APC* mutation was found to be a moderate good risk factor, with an HR of 0.65 (*χ*^2^ *P*=0.021), while tumours with *KRAS* and *TP53* mutations were in moderate poor risk, with HRs in the 1.4–1.6 range; a *BRAF* mutation, although rare (only 18/407 tumours, 4.4%), had a dramatic HR of 2.63 (in agreement with several recent reports indicating *BRAF(V600E)* as a strong negative prognostic marker of CRC^30^,^31^,^32^,^33^,^34^,^35^), while the HR was 1.05 per year over the age of 70. Adding in terms for the AKP group and for double *APC* mutation tumours (Table 5; Model 2), while allowing individual mutation effects to drop out, suggests that the increased hazard for *KRAS*- or *TP53*-mutated tumours may be largely borne by those patients with both. Including the presence of metastatic disease (Table 5; Model 3) resulted in a better fit (70 point rise in the LR *χ*^2^) and a 4.51 HR for distant metastasis. Notably, adding metastasis led to a significant HR for two-hit *APC* mutation tumours (HR=1.58, *χ*^2^ *P*=0.027, Model 3) but decreased the HR of AKP from 1.79 (*χ*^2^ *P*=0.005, Model 2) to 1.43 (*χ*^2^ *P*=0.090, Model 3) probably because of the very strong correlation of AKP with distant metastasis. Furthermore, addition of *APC*(2)KP (Class 4) that replaced AKP and two-hit *APC* mutations showed a substantially significant HR (HR=2.10, *χ*^2^ *P*=0.003; Table 5; Model 4), supporting Class 4 tumours as a worst-risk class as shown earlier in the Kaplan-Meier (KM) survival analysis (Fig. 3d).

Recently, an international consortium^36^ has coalesced six independent (gene expression) classification systems of CRCs into four consensus molecular subtypes (CMS1–4) and a fifth unclassified group (CMS_NA). We applied this system to classify 458 CRCs. The Random Forest classification (RF) and the single sample predictor (SSP) classification were used, both of which captured the mutation features of the common drivers (*APC*, *KRAS*, *TP53* and *BRAF*) similar to those reported^36^. A combined RF–SSP classification is shown in Table 6. In terms of significantly higher or lower observations than expectations, CMS1 and CMS2 showed opposite patterns for a majority of categories listed. In relationship with the five-class classification, Class 0 and Class 1 showed significantly higher or lower observations in two CMS subtypes (CMS1 and CMS2) with opposite directions (Supplementary Table 8).

Furthermore, Cox modelling of 399 MSS tumours indicated that, compared with CMS2, worse survival was significantly associated with CMS1 (HR=3.48, *χ*^2^ *P*<0.0001) and CMS_NA (HR=1.82, *χ*^2^ *P*=0.02), and was marginally associated with CMS3 (HR=1.76, *χ*^2^ *P*=0.06) but not with CMS4 (HR=1.34, *χ*^2^ *P*=0.22; Supplementary Table 9; Model 3). However, only the CMS1 tumours remained significantly associated with worse survival in the multivariable model upon adding *APC* and *BRAF* plus AKP and *APC* two-mutation (HR=2.39, *χ*^2^ *P*=0.012, Model 4) or plus *APC*(2)KP (HR=2.55, *χ*^2^ *P*=0.007, Model 5).

## Discussion

Our robust regression analysis of mutations versus amino-acid length of 468 CRC tumours objectively identified a subset of 17 genes that are commonly mutated in CRC. The striking pairwise, statistically significant, correlations of *APC*, *KRAS*, *TP53* and *BRAF* reflect a positive or negative interaction between these common drivers in CRC. These observations have shed light on a crosstalk between multiple pathways deregulated by these driver mutations, with a new focus on the role of *APC*.

Heretofore, *APC* was presumed to be an important ‘initiator' gene for the majority of CRCs^2^,^10^; thus, its newly discovered prognostic role was likely underestimated. Only with the evaluation of *APC* in the context of its partnering mutations (with *KRAS* and *TP53*) and its bi-allelic mutation status does a prognostic role emerge for *APC*, with implications for tumour evolution. Survival analysis indicates that two *APC*-truncating mutations, in the presence of mutant *KRAS* and *TP53* (AKP), carry a substantially worse prognosis than single truncating mutations, but surprisingly are equivalent to highly lethal tumours lacking any *APC* mutation. These data suggest that wild-type *APC* tumours are driven by an ‘alternate', non-WNT lethal pathway. It is noteworthy that no prognostic role for *APC* mutations was previously reported in a study of 107 CRC patients^37^. The identification of the new prognostic role of *APC* may be attributed to a more detailed mutational analysis and to a larger sample size.

While tumours with two different *APC* mutations may fit the bi-allelic theory, tumours with one mutation are of particular interest because they have improved overall survival and statistically significant different biological profiles from both *APC*wt and two-hit *APC* tumours. The WNT/β-catenin pathway was activated in these one-hit *APC* tumours as measured by IHC nuclear staining of a β-catenin antibody and expression of the 64 β-catenin-targeted genes^28^. Intriguingly, the β-catenin nuclear staining scores and the expression of the 64 targeted genes appear to be less significant in tumours with two-hit *APC* mutation or one-hit mutation plus inferred allelic loss (Fig. 2b and Supplementary Fig. 2A), suggesting a possible ‘feedback' regulation of WNT activation via non-*APC* mechanisms in these tumours. This is consistent with the notion of ‘just right' WNT/beta-catenin signalling^21^,^26^,^27^.

To interpret our observation on WNT activation in one-hit *APC* mutation tumours using the classic two-hit model of *APC* mutation in FAP, we considered several possible mechanisms as a ‘second hit'. First, allelic loss, which has been well documented to be interdependent with a single *APC* mutation^20^,^38^,^39^, and somatic recombination (Robertsonian translocation)^40^, might account for the ‘second hit' in 59 of 187 one-hit *APC* mutation samples. This is predicted by examining alternate read percentages (those mutations with percentages ⩾50% suggest such loss of heterozygosity and/or recombination); however, this methodology could not distinguish these two possible mechanisms.

Second, a secondary WNT pathway gene mutation might contribute to tumorigenicity in remaining single *APC*-mutated tumours. An analysis of the TCGA CRCs, whose whole-exome-sequencing data allowed us to examine WNT pathway genes more comprehensively, did identify a substantial number of *APC*wt (∼53%), one-hit (∼33%) or two-hit (∼30%) *APC* tumours, which had one or more non-*APC* WNT pathway gene mutations. These ‘other' WNT mutations, however, did not appear to significantly contribute to WNT/β-catenin activation. It is also noteworthy that the majority of other WNT pathway alterations were missense mutations that are predicted to be neutral or to have low functional impact. In addition, a great majority of the ‘other' WNT pathway genes are known to positively contribute to WNT pathway activation. Thus, inactivating (truncating) mutations in the WNT pathway genes might more likely suppress WNT activity rather than activate it in the majority of CRCs.

Third, methylation has been thought to play an important role in CRC. This notion is supported by the fact that the CMS1 tumours (that had a significant lower rate of *APC* mutations) show a hypermethylation profile^36^, potentially initiating tumorigenesis when *APC* mutation is absent. Hypermethylation of the *APC* promoter in CRCs has been reported previously^41^,^42^,^43^,^44^. Our analysis on the TCGA CRCs^7^ also identified a significant number of methylation events associated with the *APC* promoter, but, importantly, no statistically significant difference was observed between zero-, one- and two-hit *APC* tumours. Moreover, no significant correlation was obtained between *APC* methylation and mRNA expression. Thus, our data do not support methylation of the *APC* promoter as a ‘second hit' in one-hit *APC* tumours. This agrees with several recently published studies showing that promoter methylation in *APC* plays an insignificant role in substituting for truncating mutations^44^,^45^. Collectively, we have identified a significant number of tumours harbouring one-hit *APC* mutations, many of which retained WNT activation that could not be explained by a few possible ‘second-hit' mechanisms.

A refined survival analysis based on the *APC* mutation status and partnering mutated genes (*KRAS*, *TP53*) led us to develop a prognostic, five-class genetic classification of CRC. When all patients are considered, Class 0 tumours are significantly associated with MSI that is also strongly correlated with *BRAF* mutation. Notably, a recent report on a *BRAF(V600E)* mouse model suggests that *BRAF* may be an early driver gene for MSI-like tumours^46^. Class 0 MSS are predicted to be mesenchymal-like and are positively correlated with a ‘composite' expression signature score that predicts poorer outcome in stage 1–3 patients^29^. Notably, most of the CRC classification studies report that MSI and EMT are the only two subtypes that have been consistently shown to have a statistically significant, prognostic role^5^,^6^,^7^,^11^,^12^,^13^. For Class 4 MSS tumours, an explanation for their worse survival is not evident, as no significant difference is seen between this class and its one *APC* mutation counterpart Class 3 in terms of EMT, RAS and WNT/β-catenin expression signatures. However, we found that *APC* mRNA expression of Class 4 is significantly higher than that for Classes 1–3 and marginally significantly higher than Class 0.

It has been previously reported that *APC* expression may be associated with another function of *APC* by binding DNA polymerase β (Pol-β) and flap endonuclease 1 (Fen-1) to inhibit their DNA repair activities^9^,^47^,^48^,^49^,^50^,^51^. Since the DNA-repair-inhibitory domain of *APC* was located upstream of mutation cluster region (MCR)^9^,^49^, most two-hit *APC* mutation tumours could retain an intact DNA-repair-inhibitory domain on the allele(s) with a mutation at or downstream of the *APC* ‘mutation cluster region' (identified here as zones 3 and 4). Thus, a possible linkage of Class 4 tumours' worse survival with *APC*'s DNA-repair-inhibitory function needs to be further investigated.

Using the recently reported CMS classification system^36^, we found that CMS1, instead of CMS4 MSS tumours, were significantly associated with worse survival. This difference in CRC prognostic properties between our analysis and reported results^36^ might result from the highly heterogeneous nature of CRCs.

In conclusion, we have developed a prognostic, five-class, multigene mutation classification system for CRC, with *APC* playing a central role. We believe that routine clinical *APC* mutation assessment, in addition to other known classifiers *BRAF*, *KRAS* and *TP53*, is useful and expedient in predicting outcomes and may ultimately help improve the resolution of appropriate therapy by identifying high- versus low-risk subpopulations.

## Methods

### Tumour specimen and DNA extraction

A cohort of the 468 colorectal adenocarcinoma patients for whom primary or metastatic tissue (367 *primary* lesions from stage 1–4 patients and 101 *metastatic* lesions) was obtained between October 2006 and September 2010 and subjected to TGS was included in the study. Primary and metastatic samples were both included on the basis of our previous work, demonstrating a high degree of mutation overlap between matched primary/met samples^52^. In all cases, tissue and clinical data were collected from patients under the approval of the University of South Florida institutional review board, and informed consent was obtained from the participating patients. All the tumours were collected from curative survival resections and snap-frozen in liquid nitrogen within 15–20 min of extirpation. Tumours then underwent a macrodissection quality-control process to ensure that >80% tumour was present in the specimen that underwent sequence analysis. Normal tissue, necrotic tissue and excessive stromal tissues were dissected away from the specimen under frozen-section control. DNA was then extracted from the specimen for subsequent TGS, comprising 1,321 cancer-associated genes (Supplementary Table 1) that were selected by a joint committee (Merck & Moffitt Cancer Center).

### Independent targeted sequencing cohort

Overall, 62 primary colon adenocarcinomas (including all histologic subtypes) were profiled by Molecular Health, utilizing a comprehensive 613 gene panel (The Woodlands, TX). The assay utilizes custom hybrid-capture, full exonic sequencing on the Illumina 2500 instrument (Illumina Inc., San Diego, CA). Proprietary analytic pipelines were used to map and call variants (single-nucleotide variants, frameshift mutations and CN alterations).

The mean age of the cohort is 59.9 years, with a range of 37–89 years. The mean age of patients with zero, one and two *APC* mutations is 59.0, 58.0 and 63.4 years, respectively. Results are shown in Supplementary Data 2.

### Sequence and data analysis

A median of 15,530,823 total reads (1.40 Gbases) was generated for each sample across 1,321 genes covering 3.8 MB, which were targeted using SureSelect custom designs (Agilent Technologies Inc., Santa Clara, CA) using GAIIx sequencing technology (Illumina Inc.) by BGI (Shenzhen, China). The Burrows-Wheeler Aligner (BWA^53^) was used to align sequences to human reference hg19. The Genome Analysis ToolKit (GATK)^54^ was used for insertion/deletion realignment, quality score recalibration and variant identification. The median of average depth of coverages across samples was 140.54 × . Depth was high across the targeted region: the median percentage of targeted bases covered by ⩾10 and ⩾20 high-quality bases were 94.02% and 90.06%, respectively (Supplementary Fig. 10). ANNOVAR^55^ was used to annotate mutations. Matched normal samples were generally not available for comparison; somatic mutations were enriched by removing variants found at >1% in the 1000 Genomes project data set. In addition, 264 non-matching normal tissue samples were sequenced with the colon tumour samples, and were used to filter out normal single nucleotide variants (SNVs) and potential artefacts. Finally, data from 523 normal tissue or blood samples from the TCGA breast data set were downloaded from dbGAP and analysed using the same BWA/GATK pipeline as our sequenced samples. A total of 21,179 non-silent variants were identified as being present in normal tissues and were used for filtering suspected germline variants. Mutation counts were then based on filtered data. Mutations were manually examined with samtools tview^56^ to identify and remove suspected alignment and other artefacts. A detailed description of 22 significantly mutated genes is given in Supplementary Data 1.

### Statistical methods

Robust regression was performed using the ROBUSTREG procedure (SAS 9.1, Cary, NC), with the MM approach. A value of 3 or more was used to define outlier genes with elevated mutation rates. Associations between the presence and absence of mutations in different genes, and comparison of mutation frequencies between the Moffitt 468 data set and the TCGA data for CRC was made using the Fisher exact test. Survival curves were constructed using the Kaplan–Meier method, and the log-rank test was used to assess statistical significance. The living patients were followed for a median of 34 months. Four Cox proportional hazards models were developed to explore the prognostic significance of the four key driver genes. A piecewise linear adjustment, called Age ⩾70, which is 0 for patients <70 and linearly increasing for those 70 or more, was used to adjust for an increasing likelihood of death based on age. Model 2 adds in the effect of the AKP group (defined below) and presence of two or more *APC* truncation mutations, while model 3 shows the impact of metastatic status on the model. Both models 2 and 3 used backward elimination, with a significance-level-to-stay of 0.10 to remove nonsignificant factors. In Model 4, AKP and *APC* 2 mutations were replaced by *APC*(2)KP. A similar analysis was also carried out with CMS subtypes.

### IHC analysis of β-catenin staining

Overall, 52 FFPE samples were selected from 468 CRCs for IHC analysis using a β-catenin antibody. Briefly, the FFPE slides were stained using a Ventana Discovery XT automated system (Ventana Medical Systems, Tucson) as per the manufacturer's protocol with proprietary reagents. The mouse monoclonal antibody for β-catenin (#760-4242, Ventana, 50 tests per vial at predilute concentration) was used by a Ventana anti-mouse secondary antibody. IHC staining was analysed by a pathologist, and Allred scores were given (Supplementary Table 7).

### Expression of β-catenin-targeted genes

A set of 64 ‘consensus' β-catenin (upregulated) genes were adopted from a recent study of Herbst *et al*.^28^ A mean Log2 expression was calculated for each set of genes in *APC* subgroups. *P*-values for comparison were obtained by two-tailed, unequal variance Welch *t*-test.

### EMT and RAS signature scores

EMT and RAS signature scores were previously developed^16^,^17^ and calculated in 468 samples similarly as described in our recent report^29^.

### Analyses of TCGA CRCs

To determine the potential contribution of *APC* CN deletion, truncation, methylation and mutations of other WNT pathway genes, we have examined data from an earlier CRC publication by the TCGA consortium^7^. Level 3 data were downloaded https://tcga-data.nci.nih.gov/docs/publications/coadread_2012/. The mRNA expression was expressed by the mean of (log2 rpkm+0.00748 floor) of RNASeq data. For promoter methylation analysis, we first identified six probes at the 5′ end of the *APC* gene (Supplementary Fig. 4); five of these probes behaved similarly to each other and the methylation ratio beta values were highly correlated (range of pairwise Pearson *r*=0.964–0.978). We selected cg15020645 as a representative of this ‘promoter' group. Probe cg01240931 was located further inside the gene, and it showed a more modest correlation with the representative ‘promoter' group probe of 0.52; therefore, we examined this probe as well. We then plotted the distribution of methylation beta values in each of the *APC* ‘hit' groups. Notably, one or more mutations were observed in TCGA samples for 62 non-*APC* WNT pathway genes (out of total 65 genes adapted from Nusse's the Wnt homepage http://web.stanford.edu/group/nusselab/cgi-bin/wnt/). In addition, 10 missense and 2 truncating mutations were also observed in *RNF43*, but no mutation was seen in its closely related *ZNRF3*, both of which were recently reported to play an important role in negatively regulating WNT pathway^57^,^58^,^59^,^60^. Thus, the number of mutations for these 63 genes was applied for correlation or comparison analysis. *P*-values were obtained from the exact Wilcoxon rank-sum test.

## Additional information

**Accession codes:** The targeted exome sequencing data have been deposited in the database of Genotypes and Phenotypes (dbGaP) under accession code phs001111.v1.p1.

**How to cite this article:** Schell, M. J. *et al*. A multigene mutation classification of 468 colorectal cancers reveals a prognostic role for *APC*. *Nat. Commun.* 7:11743 doi: 10.1038/ncomms11743 (2016).

## Supplementary Material

Supplementary Information Supplementary Figures 1-10, Supplementary Tables 1-9 and Supplementary References.

Supplementary Data 1 Targeted exome sequencing data of 22 significantly mutated genes in 468 CRCs.xls

Supplementary Data 2 Targeted gene sequencing and LOH data of driver genes in 62 CRCs.xlsx

## Figures and Tables

**Table 1:** Genes with elevated non-silent mutation rates.

**Gene**	***N***†	**AA length**	**Rate‡**	**Residual scores**§			||**Codons⩾8**	¶**HRun**
				**Overall**	**MSI**	**MSS**		
*KRAS*	192	189	72.4	13.52	3.46	14.09	4	0
*TP53*	292	393	52.9	16.25	3.67	17.1	6	0/1
HLA-A*	102	365	19.9	8.52	3.91	8.43	1	0
*APC*	494	2,843	12.4	17.08	—	18.75	9	15/18
*SMAD4*	59	552	7.6	5.16	—	5.68	1	0
*FBXW7*	62	707	6.2	4.92	3.12	4.54	2	0/1
MUC4*	463	5,412	6.1	13.30	4.72	14.06	5	0/3
*BRAF*	61	766	5.7	4.69	5.35	—	1	0/1
*TCF7L2*	48	602	5.7	4.16	—	4.32		11/32
*PIK3CA*	76	1,068	5.1	4.97	—	4.82	3	0
*GNAS*	62	1,037	4.3	4.09	—	3.77		0
*TAF1L*	70	1,826	2.7	3.09	—	—		0/9
*CSMD3*	115	3,707	2.2	3.06	—	—		0/6
*LRP1B*	138	4,599	2.1	3.19	—	4.02		1/3
OBSCN*	220	7,968	2.0	3.49	—	3.56		0/7
SYNE1*	227	8,797	1.8	3.08	—	4.01		0/1
TTN*	845	34,350	1.8	5.30	—	6.76		3/23
CBX4*	31	560	3.9	—	3.10	—	1	0/5
ITGB4*	54	1,822	2.1	—	3.71	—		0/4
*ADAMTS18*	52	1,210	3.1	—	—	3.08		0/19
*FAM123B*	41	1,135	2.6	—	—	3.13		0/5
MUC16*	295	14,507	1.4	—	—	3.78		0/3

AA, amino acid; GATK, Genome Analysis ToolKit; MSI, microsatellite instability; TCGA, The Cancer Genome Atlas.

†*N*=number of non-silent mutations. ‡Rate=10^5^ × *N*/(468(3 × AA length)). §Residual scores denote the *z*-score for difference of the mutation count from the fitted robust regression line (SAS 9.3). Residual scores ⩾3 are considered to be outliers. A larger score represents a more substantial difference. ||Codons ⩾8=No. of codons with mutations in ⩾8 tumours. For example, *KRAS* has four codons with a mutation in at least eight tumours (12, 13, 61 and 146). ¶HRun=MSS-associated/total homopolymer run mutations: genomic loci where a single-nucleotide base occurs numerous times consecutively in the human reference, as determined by GATK. *APC* and *BRAF* counts include all non-silent mutations as well. However, we include only *APC*-truncating and *BRAF* (V600E) mutations (that are considered as clearly functional) in the further analysis. See detailed sequencing data in Supplementary Data 1.

^*^denotes genes with significantly lower non-silent mutation rates in TCGA samples.

**Table 2:** Percentile ranking of Spearman correlation among 1,082 genes.

**Gene mutation**	**MSI-H**	**Distant-Mets**	**Gene mutation**				**Gene signature**	
			***APC***	***TP53***	***KRAS***	***BRAF***	**EMT**	**RAS**
*APC*	**0.1**	**99.6**	—	**100**	**100**	**0.1**	23	**0.2**
*TP53*	**0.2**	**99.4**	**99.9**	—	**6**	**0.4**	74	**0.1**
*KRAS*	**0.3**	**100**	**100**	18	—	**0.2**	**2**	**100**
*SMAD4*	**4**	**95**	84	85	**93**	52	13	89
*TCF7L2*	22	58	**98**	67	70	**8**	49	29
*FAM123B*	36	13	**97**	31	**97**	22	71	80
*GNAS*	49	69	60	67	86	71	28	**98**
*ADAMTS18*	51	51	40	56	69	50	38	56
*LRP1B*	66	81	45	91	38	80	55	58
*PIK3CA*	67	28	85	**7**	**98**	32	**1.4**	51
*TAF1L*	81	48	39	80	21	**93**	24	84
*FBXW7*	86	12	63	56	38	78	41	54
*SYNE1*	**93**	21	**4**	30	50	**95**	24	**99**
*CSMD3*	**93**	34	**7**	48	62	**99**	**6**	**99.4**
*CBX4*	**95**	**7**	**3**	43	**2**	**98**	56	50
*ITGB4*	**99.9**	17	**0.8**	11	46	**99.6**	43	**99.7**
*BRAF*	**100**	**3**	**0.1**	16	**0.1**	—	37	**99.9**

Only genes with Z5 non-silent mutations were analysed. APC, Adenomatous polyposis coli; EMT, epithelial–mesenchymal transition; Mets, metastasis; MSI, microsatellite instability.

(1) Closer examination of the literature and our own data suggest that only the *BRA*F(V600E) (comprising 53/60 patients with BRAF mutations) and *APC*-truncating mutations (stopgains or frameshift mutations, as these mutations clearly inactivate downstream β-catenin-binding sites) should be considered as clearly functional for these two genes; hereafter, we include only *BRAF*(V600E) and *APC*-truncating mutations in the further analysis and simply refer to these specific mutations as *BRA*F and *APC* mutations.

(2) MSI-H is a binary variable for the MSI-H status.

(3) EMT and RAS refer to published gene signature scores^16^,^17^.

(4) Percentiles ≤5 or ⩾95 are shown in **bold**.

**Table 3:** *APC* truncation mutation profiles.

No. of *APC* Mutations*						
**Zones 1–2**	**Zones 3–4**	***N***	**MSI**	**Loss**	**Allelic WNT**^**†**^	**Other remaining**
0	0	156	45	0	36	75
0	1	130	3	46	35	44
1	0	57	4	13	17	24
2	0	7^‡^	2	0	3	2
0	2	8	0	2	2	6
1	1	105	6	8	22	67
1	2–3	2^§^	0	0	2	0
2	1	3^§^	1	0	1	1
Total		468	61	69	118	219

*APC, Adenomatous polyposis coli*; Mets, metastasis; MSI, microsatellite instability.

*zones 1–2 include *APC* codons 1–1,262; zones 3–4 include *APC* codons 1,263–2,843. Also see Supplementary Table 5.

†The mutations were tabulated only for cases that had neither MSI nor allelic loss. Other WNT pathway mutations included the following: *CTTNA1, CTNNB1, CTTND2, WNT1, WNT2, WNT2B, WNT4, WNT9B, WNT10B, AMER1, TCF7L2, MACF1, AXIN1, WIF1, GSK3B* and *CDH1.*

‡In all seven samples, one of the mutations was in a variant isoform of *APC* (not the canonical isoform NM_0001127511), which we believe does not violate the prevailing theory that at least one functional 20-amino-acid β-catenin-binding site^27^ is needed, as we expect that the canonical APC isoform will sometimes be translated into protein by the tumour, which have some functional binding sites. This is consistent with the reduced impact of other *APC* alternative splice-variant mutations^61^.

§These five samples have more than the two truncation mutations. Thus, at least two of the *APC* mutations reside on the same allele. Again, consistent with the prevailing theory, we infer that the three cases with two zone 1–2 hits reside on the same allele and two of these cases are hypermutated *POLE* tumours.

**Table 4:** Five classes grouped by the *APC* mutation status with partnering mutations.

**Classes**	**Age**		**%Right**	**%MSI**	**%Mets**	**% Stage**			**Devel_Mets**
	***N***	**Median**				**1–2**	**3**	**4**	
0 ‘*APC*wt'	156	70	53 ↑	29 ↑↑↑	29	49	29	21	8
1* ‘A(1), A(1)K, A(1)P'	142	64	28 ↓	5 ↓↓	37	42	32	24	13
2^†^ ‘A(2), A(2)K, A(2)P'	84	65	37	10	25	50	27	21	4 ↓
3^‡^ ‘*APC*(1)KP'	45	63	40	0 ↓	51	31	40	27	24 ↑↑
4§ ‘*APC*(2)KP'	41	66	51	2	51	15 ↓↓	54 ↑	32	20
Trend *p*-value	468		0.090	<0.0001	0.015		0.009		0.031

*APC, Adenomatous polyposis coli*; Mets, metastasis; MSI, microsatellite instability.

(1)* A−*APC*, K−*KRAS*, P−*TP53*.

Class 0: *APC* wild type; Class 1: *APC(1), APC(1)*/*KRAS*, *APC(1)*/*TP53*; Class 2: *APC(2), APC(2)*/*KRAS*, *APC(2)*/*TP53*; Class 3: *APC(1)/KRAS*/*TP53*; Class 4: *APC(2)/KRAS*/*TP53.*

While A(1) or *APC* (1) represents one *APC* mutation, A(2) or *APC* (2) represents two *APC* mutations.

(2)† There is significantly higher or lower observation than expectation: ↑(↓) for *P*<0.05; ↑↑(↓↓) for *P*<0.01; ↑↑↑(↓↓↓) for *P*<0.001, based on individual *χ*^2^ contribution from the table cell.

(3)‡ Devel_Mets for the patient who was initially diagnosed with primary cancer (not stage 4) and developed Mets after that.

(4)§ A significantly higher (than expectation) rate of MSI was observed in Class 0 (*χ*^2^, *P*<0.001), while the lower rate was associated with one *APC*-mutation tumour (Class 1, *χ*^2^, *P*<0.01 and Class 3, *χ*^2^, *P*<0.05). Class 4 tumours were significantly associated more with stage 3 (*χ*^2^, *P*<0.05) and less with stage 1–2 tumours (*χ*^2^, *P*<0.01). However, a higher rate of ‘developing' metastasis (Devel_Mets) was demonstrated for Class 3 (*χ*^2^, *P*<0.01).

**Table 5:** Cox model predictors of overall survival of MSS tumours (*N*=407).

**Covariable**	**N**	**Model 1**		**Model 2**		**Model 3**		**Model 4**	
		**HR**	***P*****-value**	**HR**	***P*****-value**	**HR**	***P*****-value**	**HR**	***P*****-value**
*APC*	296	0.65	0.021	0.56	0.008	0.49	0.001	0.59	0.006
*KRAS*	179	1.62	0.007						
*TP53*	261	1.41	0.056						
*BRAF*	18	2.63	0.002	2.38	0.005	2.23	0.011	2.25	0.011
AKP	85			1.79	0.005	1.43	0.090		
Two *APC* mut	116			1.29	0.217	1.58	0.027		
*APC*(2)KP	40							2.10	0.003
Metastasis	155					4.55	<0.0001	4.49	<0.0001
Age (⩾70)	131	1.05	0.005	1.04	0.008	1.09	<0.0001	1.09	<0.0001
LR *χ*2	407	29.7	<0.0001	28.9	<0.0001	98.5	<0.0001	97.9	<0.0001

*APC, Adenomatous polyposis coli*; HR, hazards ratio; LR, likelihood ratio.

**Table 6:** CMS classification of 458 CRCs.

**RF_SSP**	***N***	APC*	***APC*****1 mut**	***APC*****2 mut**	***APC*****_ LOH**	***KRAS***	***TP53***	***TP53*****LOH**	***BRAF***	**MSI_H**	**Right**	**D_meta**
CMS1^†^	77	32 ↓↓^‡^	18 ↓↓	14	1 ↓↓	22 ↓↓	44	13 ↓^§^	52 ↑↑	62 ↑↑	68 ↑↑	23
CMS2	116	90 ↑↑^||^	59 ↑↑	31	28 ↑↑	29 ↓	85 ↑↑	55 ↑↑	0 ↓↓	0 ↓↓	19 ↓↓	43
CMS3	64	72	36	36	22	73 ↑↑	39 ↓	23	5	8	48	14 ↓↓
CMS4	112	63	42	21	6	42	56	16 ↓	3 ↓↓	1 ↓↓	40	51 ↑↑
CMS_NA^¶^	89	70	45	25	8	51	61	20	7	6	44	29
Total^#^	458	307	192	115	61	190	275	125	52	59	188	160

*APC, Adenomatous polyposis coli*; CMS, consensus molecular subtype; CRC, colorectal cancer; MSI, microsatellite instability.

This table shows the distribution of four drivers, MSI status and distant metastasis (D_meta).

*APC represents all *APC* mutation statuses including *APC* 1 mut (one truncated mutation), *APC* 2 mut (two truncated mutations) and *APC*_LOH (inferred allelic loss).

Number in the table represents the per cent of interested observation in each CMS category, for example, 90% of CMS2 had *APC* mutations and 62% of CMS1 were MSI_H cases.

#Number in the ‘Total' row represents the number of cases.

There is significantly higher observation than expectation (||↑↑: *P*<0.01), based on individual *χ*^2^ contribution from the table cell.

There is significantly lower observation than expectation ((§↓: *P*<0.05; ‡↓↓: *P*<0.01), based on individual *χ*^2^ contribution from the table cell.

†The frequencies of MSI and *BRAF* mutation were higher in CMS1 but lower in CMS2, and, by contrast, *APC* mutations and *TP53* ‘LOH' were seen in more CMS2 but were fewer in CMS1. Moreover, the CMS3 subtype was significantly associated positively with *KRAS* mutation, but negatively correlated with the rate of ‘developing' metastasis, while CMS4 tumours had fewer MSI but more ‘developing' metastasis cases.

¶CMS_NA, unclassified samples that were not applicable to CMS1-4.
