# Supplementary material for: A multigene mutation classification of 468 colorectal cancers reveals a prognostic role for APC
Source: Nat Commun. 2016 Jun 15;7:11743. doi: 10.1038/ncomms11743 (PMC4912618; doi:10.1038/ncomms11743)
Supplement: Supplementary Information — Supplementary Figures 1-10, Supplementary Tables 1-9 and Supplementary References. [file ncomms11743-s1.pdf]

SUPPLEMENTARY FIGURES

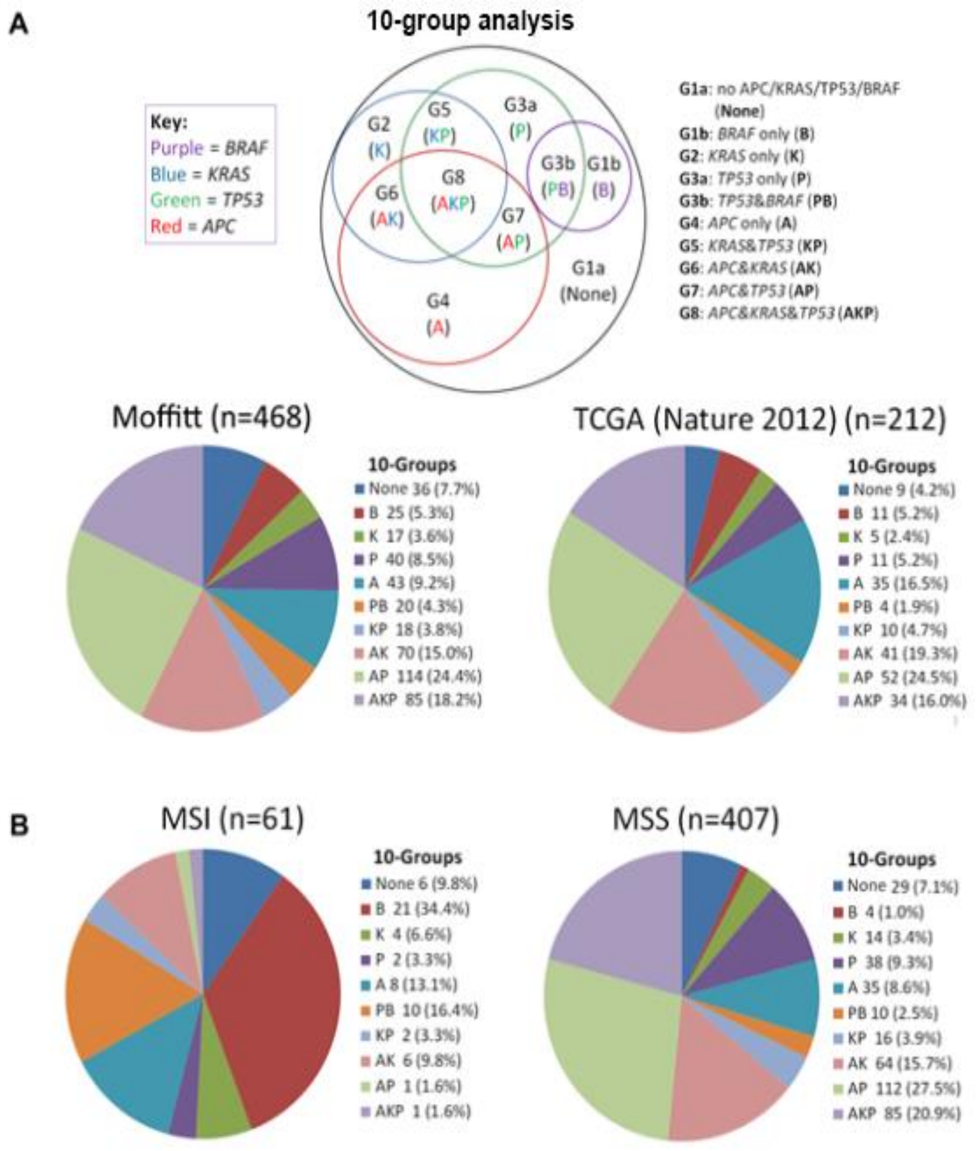

Supplementary Figure 1. The 10-group analysis of colorectal cancer. 10 groups were formed according to the pairwise, statistically significant, positive or negative correlations between somatic mutations of four driver genes *APC*, *KRAS*S, *TP53* and *BRAF*. Presence or absence of *APC*, *KRAS*, and *TP53* mutations would define eight possible combinatorial groups; *BRAF*-mutated tumors only comprise two additional significant groups (i.e. *BRAF* only and *TP53* &

BRAF), given its strong anti-associations with *KRAS* (with which it did not co-occur), and *APC*. Notably, a significant relationship between *BRAF* and *TP53* was also reported by another group [1]. "None" – a group of tumors not having the four driver mutations in our analysis. **(A)** The distribution of *APC*, *KRAS*, *TP53* and/or *BRAF* mutations in each group in the Moffitt (n = 468) and TCGA (n = 212) [2] datasets. **(B)** The mutation distribution in each group in the Moffitt MSI (n = 61) and MSS (n = 407) tumors.

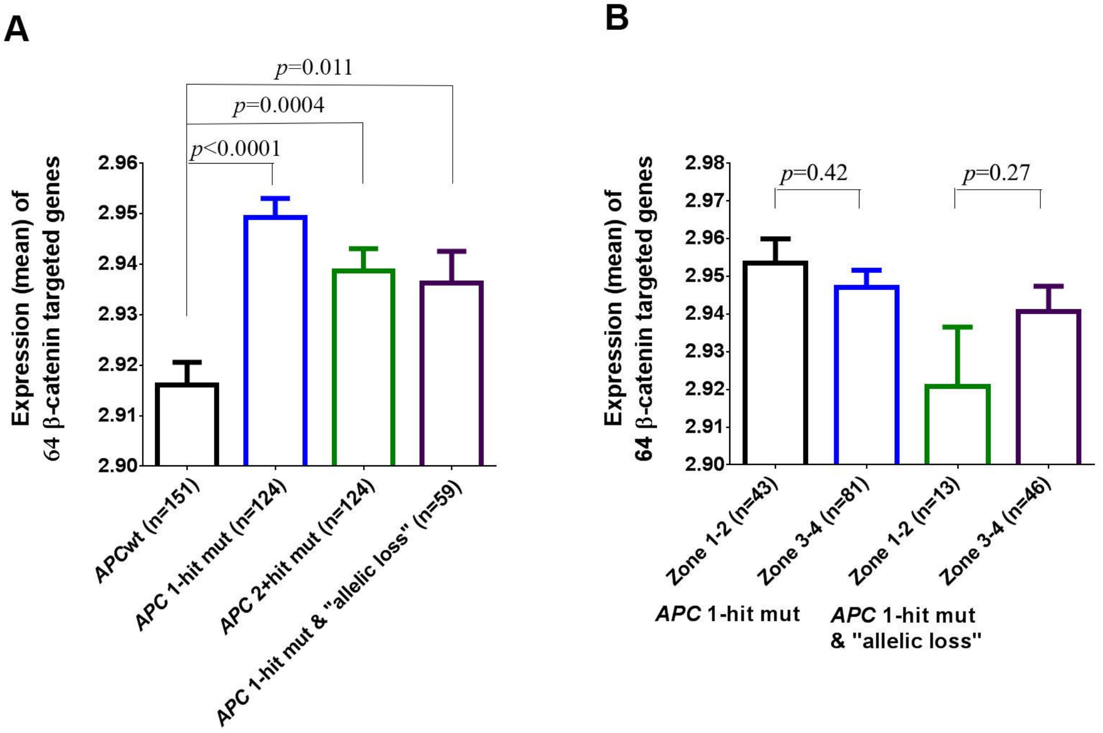

Supplementary Figure 2. Expression of β-catenin targeted genes in *APC* groups (n=458, 10 samples without suitable microarray data were excluded from 468 CRCs). (**A**) The mean mRNA (log2) expression of 64 β-catenin targeted genes [3] relative to each of four *APC* subgroups. (**B**) Comparison of expression of WNT targets between zone 1-2 mutations and zone 3-4 mutations in *APC* one mutation tumors without or with inferred allelic loss. Error bars represent standard errors (SEM). *p* values were obtained from two-tailed, unequal variance Welch *t* test.

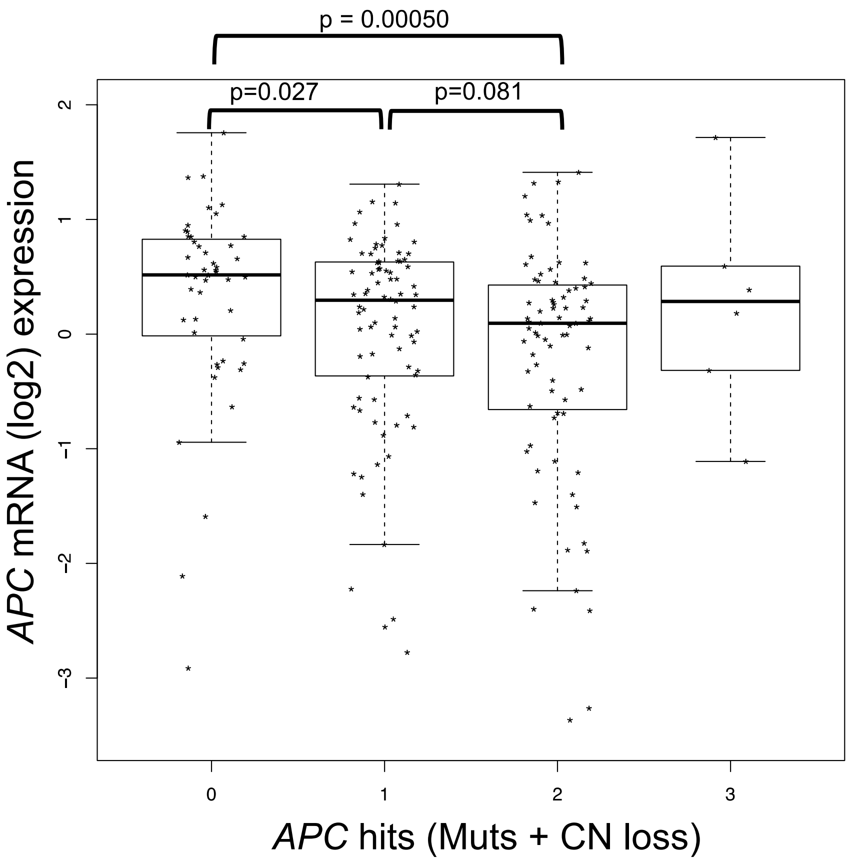

Supplementary Figure 3. ***APC*** **mRNA expression by derived hits in TCGA CRCs** [2] for which 209 samples had data of mutation, CNA and RNAseq expression. The mean of log2 rpkm was used for mRNA expression of *APC*. CN loss represents copy number loss. *p* values were obtained by the exact Wilcoxon rank sum test. The bolded horizontal lines indicate the median value, the top and bottom box edge indicate the third and first quartiles, respectively, and the whiskers indicate the most extreme data point no more than 1.5 times the interquartile range.

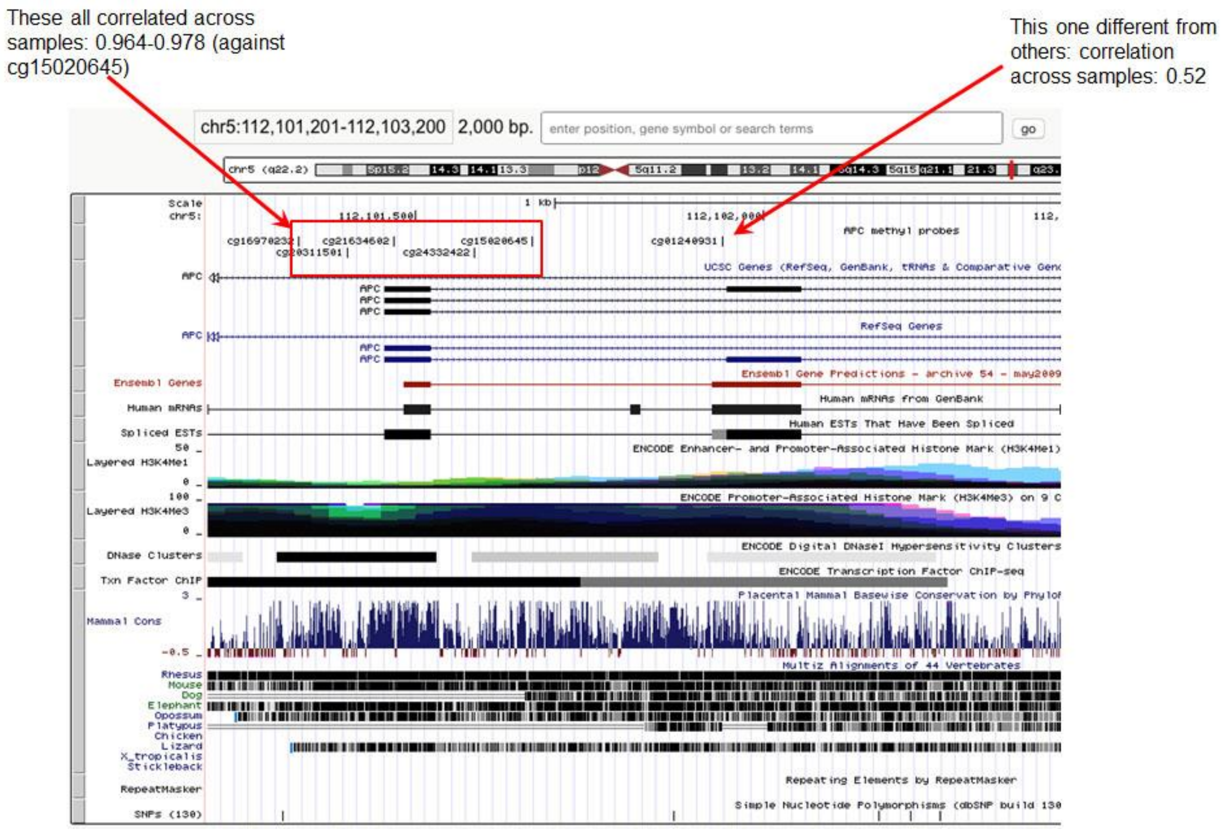

Supplementary Figure 4. ***APC* methylation probes used in TCGA CRCs [2].**

A UCSC Genome browser (http://genome.ucsc.edu/) illustrating the location of 6 APC methylation probes relative to the gene model itself. Note that 5 probes localize to the gene start, and 1 probe is localized further into the gene body.

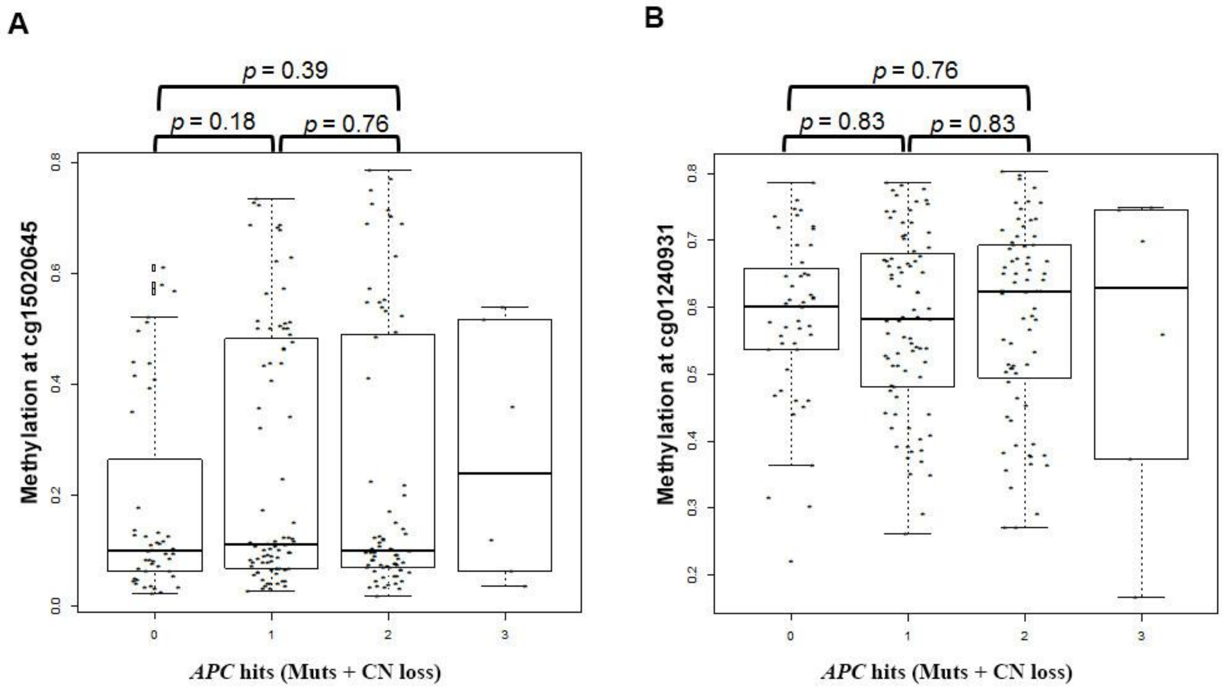

Supplementary Figure 5. ***APC*** **methylation by derived hits in TCGA CRCs** [2]. (**A**) *APC* probe cg15020645 (promoter region). (**B**) *APC* probe cg01240931 (within gene). CN loss represents copy number loss. *p* values were obtained by exact Wilcoxon rank sum test. The bolded horizontal lines indicate the median value, the top and bottom box edge indicate the third and first quartiles, respectively, and the whiskers indicate the most extreme data point no more than 1.5 times the interquartile range.

A.

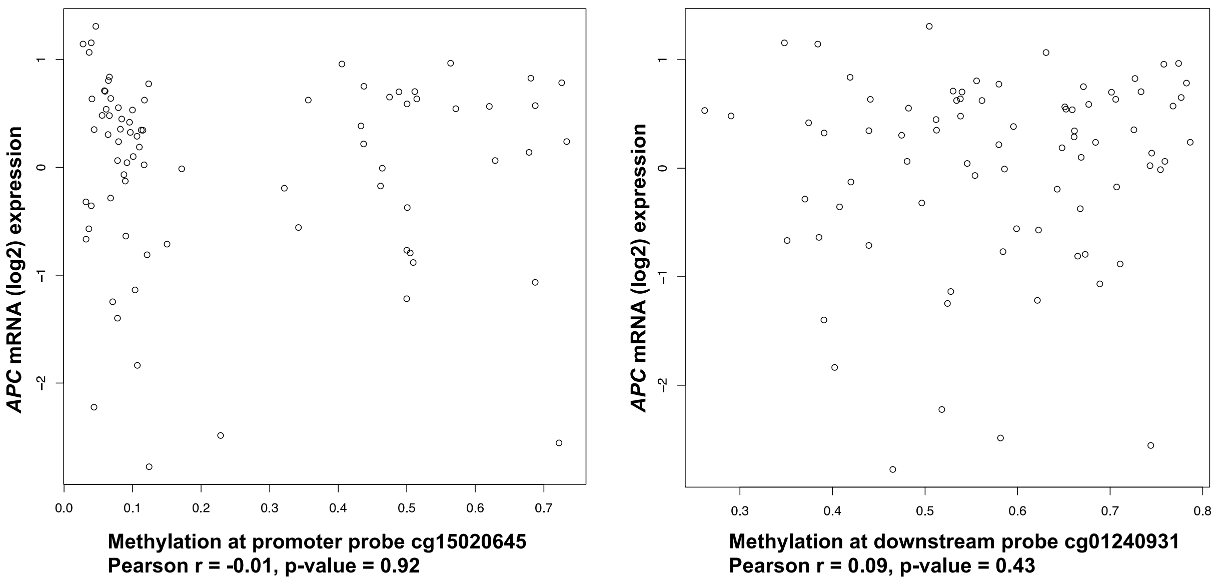

B.

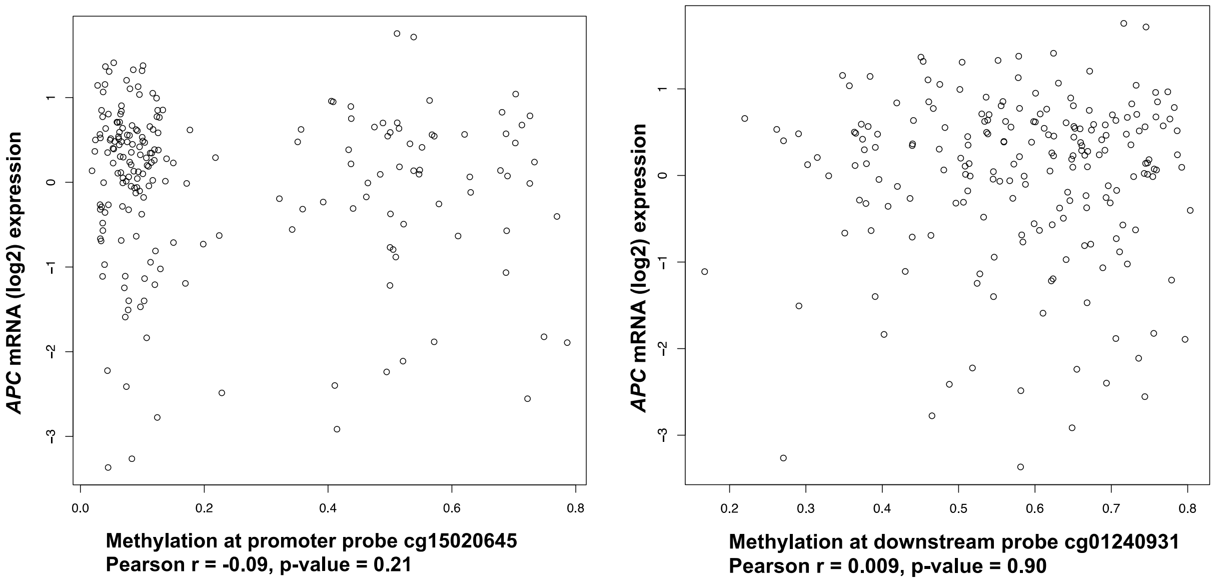

Supplementary Figure 6. *APC* expression versus methylation in TCGA CRCs [2].

(A) 1-hit samples. (B) All samples. No significant correlation was observed for either probe in either 1-hit or all samples.

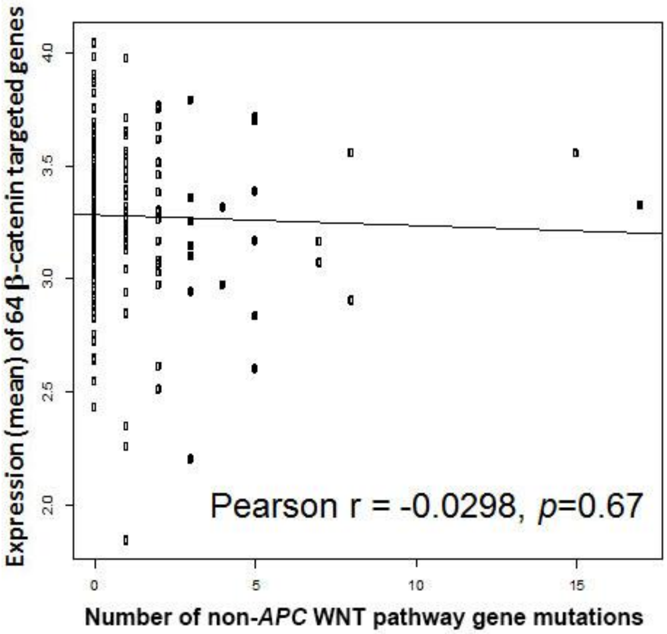

Supplementary Figure 7. Correlation of the expression of the 64 β-catenin targeted genes [3] versus the number of non-*APC* WNT pathway gene mutations (63 genes examined) in TCGA CRCs [2]. The mean of log2 rpkm was used for mRNA expression of the 64 genes.

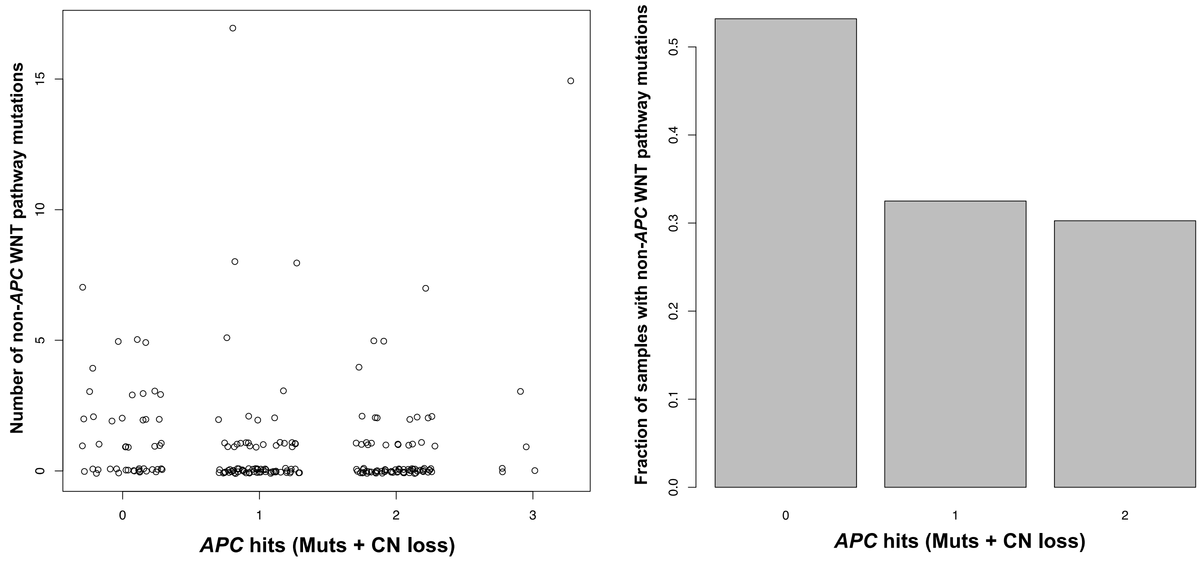

Supplementary Figure 8. non-*APC* WNT pathway gene mutations (63 genes examined) across derived hits in TCGA CRCs [2]. Left: Number of non-*APC* WNT pathway mutations observed in each *APC* hit class. Right: Fraction of samples with non-*APC* WNT pathway mutations in each *APC* hit class.

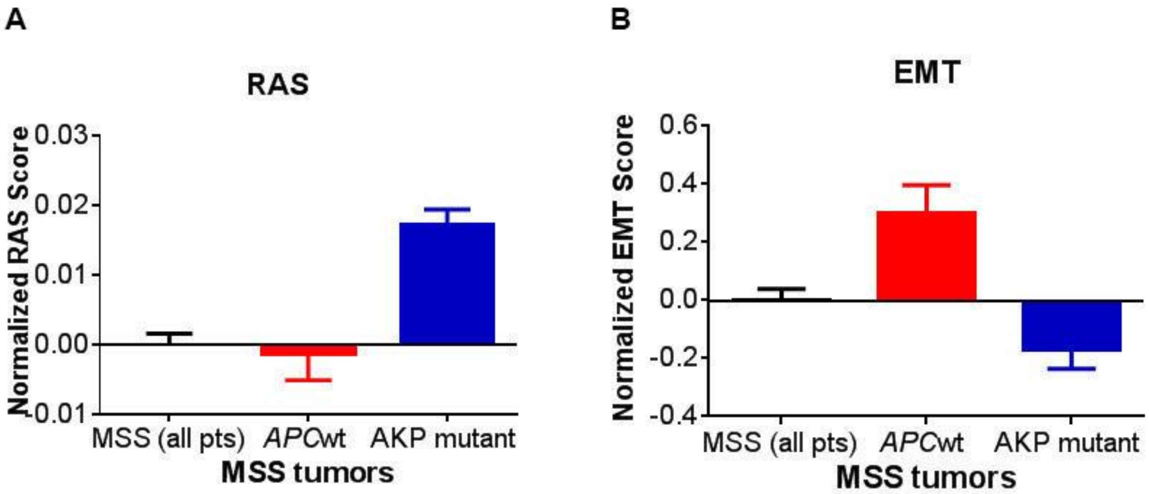

Supplementary Figure 9. Comparison of the RAS_AZ [4] (A) or EMT [5] (B) signature scores between MSS tumors containing wild type *APC* (n=156) and the AKP (*APC*, *KRAS*, *TP5*3) triple mutant (n=85). The scores were normalized by the average scores of all 407 MSS tumors (from total 468 all patient samples) which were used as references and set to be zero. Error bars represent standard errors (SEM). The two-tailed, unequal variance Welch *t* test was used to assess the statistical significance of comparison, p<0.0001 for both RAS/MAKP and EMT scores as compared between *APC*wt and AKP mutant groups.

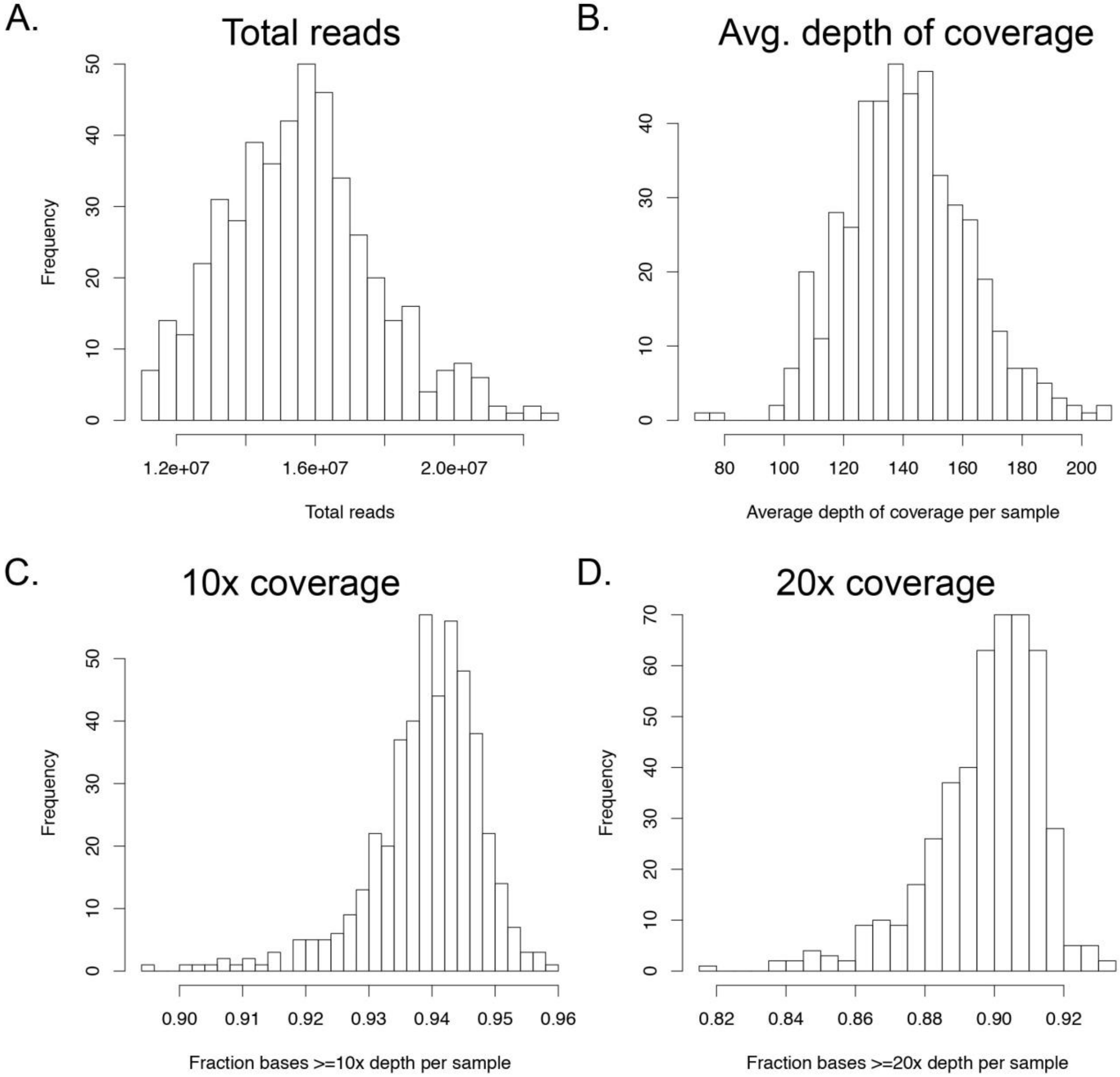

Supplementary Figure 10. Sequencing metrics. (**A**) Distribution of total sequenced reads across sample. (**B**) Distribution of average final depth of coverage. (**C**) Distribution of fraction targeted bases covered by >=10 reads (**D**) >=20 reads.

SUPPLEMENTARY TABLES (Supplementary Tables 2 and 8 are attached as separate EXCEL files)

Supplementary Table 1. List of 1321 genes in targeted gene sequencing

ABCA1	*ABCA3*	*ABCB1*	*ABCC2*	*ABCC3*	*ABCC4*	*ABL1*	*ABL2*
ACAP1	*ACVR1B*	*ACVR2A*	*ADA*	*ADAM17*	*ADAM29*	*ADAM33*	*ADAMTS18*
ADAMTS20	*ADAMTSL3*	*ADCY1*	*ADCY9*	*ADH7*	*ADHFE1*	*ADORA2A*	*ADORA2B*
ADRA1A	*ADRB3*	*AFF4*	*AKAP6*	*AKAP9*	*AKT1*	*AKT1S1*	*AKT2*
AKT3	*ALK*	*ALOX12B*	*ALOX15*	*ALPK2*	*ALPK3*	*ALS2*	*ANAPC5*
ANKRD32	*ANO1*	*APAF1*	*APBB1IP*	*APC*	*APC2*	*APEX1*	*APOB*
AR	*ARAF*	*ARAP3*	*AREG*	*ARFRP1*	*ARHGAP26*	*ARHGAP29*	*ARHGAP6*
ARHGEF11	*ARHGEF12*	*ARID1A*	*ARID3A*	*ARNT*	*ARRB1*	*ASXL1*	*ATG10*
ATG12	*ATG16L1*	*ATG16L2*	*ATG2B*	*ATG4D*	*ATG5*	*ATG9B*	*ATM*
ATOH1	*ATP8B1*	*ATR*	*ATRX*	*AURKA*	*AURKB*	*AURKC*	*AXIN1*
AXIN2	*AXL*	*AZI2*	*BAD*	*BAI1*	*BAI2*	*BAI3*	*BAK1*
BAP1	*BARD1*	*BAT1*	*BAX*	*BAZ1A*	*BBC3*	*BCAT1*	*BCL10*
BCL11A	*BCL2*	*BCL2A1*	*BCL2L1*	*BCL2L11*	*BCL2L14*	*BCL2L15*	*BCL2L2*
BCL3	*BCL6*	*BCL9*	*BCL9L*	*BCORL1*	*BCR*	*BECN1*	*BID*
BIK	*BIRC2*	*BIRC3*	*BIRC5*	*BIRC6*	*BIRC7*	*BLM*	*BMF*
BMI1	*BMP2*	*BMP4*	*BMPR1A*	*BMPR1B*	*BMPR2*	*BMX*	*BNIP3*
BOK	*BPTF*	*BRAF*	*BRCA1*	*BRCA2*	*BRD2*	*BRD3*	*BRIP1*
BTG2	*BTK*	*BTRC*	*BUB1*	*C10orf137*	*C12orf63*	*C1orf144*	*C9orf96*
CA12	*CA9*	*CABLES1*	*CAD*	*CALCR*	*CALCRL*	*CARD11*	*CASC5*
CASK	*CASP2*	*CASP8*	*CASP9*	*CAV1*	*CBFA2T3*	*CBL*	*CBLB*
CBLC	*CBX4*	*CBX7*	*CBX8*	*CCBP2*	*CCDC63*	*CCDC82*	*CCKAR*
CCKBR	*CCL18*	*CCL2*	*CCL8*	*CCNA1*	*CCNA2*	*CCND1*	*CCND2*
CCND3	*CCNDBP1*	*CCNE1*	*CCNE2*	*CCR3*	*CCR5*	*CD14*	*CD22*
CD248	*CD33*	*CD34*	*CD4*	*CD44*	*CD79A*	*CD79B*	*CD8A*
CDC25A	*CDC25B*	*CDC25C*	*CDC42*	*CDC42BPA*	*CDC42BPB*	*CDC6*	*CDC7*
CDC73	*CDH1*	*CDH11*	*CDH2*	*CDH20*	*CDH5*	*CDK1*	*CDK12*
CDK19	*CDK2*	*CDK4*	*CDK5*	*CDK6*	*CDK7*	*CDK8*	*CDK9*
CDKL2	*CDKN1A*	*CDKN1B*	*CDKN1C*	*CDKN2A*	*CDKN2B*	*CDKN2C*	*CDKN2D*
CDKN3	*CDS1*	*CEBPA*	*CELSR1*	*CELSR2*	*CENPF*	*CEP110*	*CES3*
CHD5	*CHD8*	*CHEK1*	*CHEK2*	*CHIC2*	*CHKA*	*CHKB*	*CHRNA3*
CHRNA5	*CHRNB4*	*CHUK*	*CIC*	*CLSPN*	*CLTC*	*CLU*	*CMAS*
CNTD2	*CNTN1*	*COL11A1*	*COL14A1*	*COL1A1*	*COL7A1*	*CREBBP*	*CRHR1*
CRKL	*CRLF2*	*CSDA*	*CSF1R*	*CSK*	*CSMD3*	*CSNK1G2*	*CTDP1*
CTNNA1	*CTNNB1*	*CTNND2*	*CTSH*	*CUBN*	*CX3CR1*	*CXCR4*	*CXorf30*
CYB5D2	*CYLD*	*CYP1B1*	*CYP2C19*	*CYP2C8*	*CYP2D6*	*CYP3A4*	*CYP3A5*
DAB2	*DAXX*	*DBF4*	*DBF4B*	*DBN1*	*DCC*	*DCLK3*	*DCLRE1C*
DDB1	*DDB2*	*DDIT3*	*DDR1*	*DDR2*	*DDX1*	*DDX10*	*DDX11*
DGKA	*DGKB*	*DGKG*	*DGKZ*	*DIP2C*	*DIS3*	*DKK1*	*DLC1*
DLG1	*DLL1*	*DLL3*	*DLL4*	*DNAH8*	*DNAJC24*	*DNMT1*	*DNMT3A*
DNMT3B	*DNTT*	*DOK1*	*DOT1L*	*DPYSL4*	*DTX1*	*DUSP1*	*DUSP10*
DUSP14	*DUSP2*	*DUSP4*	*DUSP5*	*DUSP6*	*DUSP9*	*DYRK2*	*E2F1*
E2F3	*E4F1*	*ECT2*	*EEF2K*	*EGF*	*EGFL6*	*EGFR*	*EGLN1*
EIF2AK1	*EIF4E*	*ELK1*	*ELK3*	*ELP2*	*ENPP2*	*EP300*	*EP400*
EPHA1	*EPHA10*	*EPHA2*	*EPHA3*	*EPHA4*	*EPHA5*	*EPHA6*	*EPHA7*
EPHA8	*EPHB1*	*EPHB2*	*EPHB3*	*EPHB4*	*EPHB6*	*EPO*	*ERBB2*
ERBB3	*ERBB4*	*ERC1*	*ERCC2*	*ERCC3*	*ERCC4*	*ERCC5*	*ERCC6*
EREG	*ERG*	*ERGIC3*	*ERN1*	*ERN2*	*ERRFI1*	*ESR1*	*ESR2*
ETS1	*ETS2*	*ETV1*	*ETV4*	*ETV5*	*ETV6*	*EVC2*	*EWSR1*
EXOC2	*EXOC4*	*EXT1*	*EXT2*	*EZH2*	*F2RL2*	*FADD*	*FAM123B*
FAM46A	*FANCA*	*FANCB*	*FANCC*	*FANCD2*	*FANCE*	*FANCF*	*FANCG*
FANCL	*FANCM*	*FAS*	*FASLG*	*FASN*	*FBXL6*	*FBXO10*	*FBXO32*
FBXW7	*FCGR3A*	*FEN1*	*FER*	*FES*	*FGF10*	*FGF19*	*FGF2*
FGF3	*FGF4*	*FGFR1*	*FGFR2*	*FGFR3*	*FGFR4*	*FH*	*FHIT*
FIGF	*FKTN*	*FLI1*	*FLNB*	*FLT1*	*FLT3*	*FLT4*	*FN1*
FOS	*FOSL1*	*FOXL2*	*FOXM1*	*FOXO1*	*FOXO3*	*FOXP3*	*FOXP4*
FPR3	*FRMD7*	*FSCB*	*FSCN1*	*FURIN*	*FUT4*	*FYN*	*FZD10*
G3BP2	*GAB1*	*GAB2*	*GAB3*	*GABARAPL1*	*GABPA*	*GADD45G*	*GATA1*
GEN1	*GLI1*	*GLI3*	*GLP1R*	*GLS*	*GNA11*	*GNAQ*	*GNAS*
GOLIM4	*GPC5*	*GPR124*	*GPR81*	*GPR84*	*GRB10*	*GRB2*	*GRIN2A*

GRK5	GRM1	GRM3	GRM5	GRM6	GRM7	GRM8	GSK3A
GSK3B	GSPT1	GSTP1	GSTT1	GSX2	GTPBP4	GUCY1A2	GUCY2F
HAPLN1	HCCS	HCRTR2	HDAC1	HDAC2	HDAC4	HDAC9	HDLBP
HECW1	HECW2	HERC1	HERC3	HERC6	HES1	HES4	HES5
HES6	HES7	HEY1	HEY2	HEYL	HGF	HIF1A	HIP1
HIPK2	HLA-A	HMGA1	HMGA2	HNF1A	HOXA3	HOXA9	HOXD13
HRAS	HRK	HSD11B1	HSD17B2	HSF1	HSP90AA1	HSP90AB1	HSPA2
HUS1	ICK	ID1	IDH1	IDH2	IGF1	IGF1R	IGF2
IGF2R	IGFBP1	IGFBP2	IGFBP3	IGFBP4	IGFBP5	IGFBP6	IGFBP7
IGFBPL1	IKBIP	IKBKAP	IKBKB	IKBKE	IKBKG	IKZF1	IKZF3
IL12A	IL12B	IL1B	IL21R	ILF3	ILK	ING4	INHBA
INPP4A	INPP4B	INPP5A	INPPL1	INSR	INSRR	IQGAP1	IRAK2
IRAK3	IRAK4	IRF4	IRS1	IRS2	IRS4	ITCH	ITGA10
ITGA9	ITGAL	ITGAV	ITGB2	ITGB3	ITGB4	ITK	ITPA
ITPR1	ITPR2	ITPR3	ITSN2	JAG1	JAG2	JAK1	JAK2
JAK3	JUN	JUNB	KAT2B	KCNH8	KDM1A	KDM4C	KDM5A
KDM5C	KDM6A	KDR	KEAP1	KIAA0182	KIAA1409	KIF16B	KISS1
KIT	KLF6	KNTC1	KRAS	KSR1	KSR2	KTN1	LAMC1
LAMP1	LARGE	LATS1	LATS2	LCK	LCP1	LDHA	LDHB
LEF1	LGALS7	LGR5	LHCGR	LIG3	LIG4	LMO2	LPHN1
LPHN2	LPHN3	LRP1	LRP1B	LRP5	LRP6	LRRC10	LRRC7
LRRK2	LRRN3	LTA	LTBP1	LTBR	LTF	LTK	LYN
MACF1	MAG	MAGI1	MAGI2	MAGI3	MAMDC4	MAN1B1	MAP1LC3A
MAP1LC3B	MAP1LC3C	MAP2K1	MAP2K2	MAP2K4	MAP2K6	MAP2K7	MAP3K1
MAP3K11	MAP3K14	MAP3K2	MAP3K5	MAP3K6	MAP3K8	MAP3K9	MAP4K4
MAPK1	MAPK10	MAPK13	MAPK3	MAPK7	MAPK8	MAPK8IP2	MAPK8IP3
MAPK9	MAPKAP1	MAPT	MARK1	MARK4	MAS1	MAS1L	MAST4
MCHR2	MCL1	MCM3AP	MCPH1	MDC1	MDK	MDM2	MDM4
MECOM	MEF2C	MELK	MEN1	MERTK	MET	MEX3B	MGA
MGC42105	MGMT	MICAL1	MINK1	MITF	MKNK1	MKNK2	MKRN3
MLH1	MLH3	MLL	MLL2	MLL3	MLL4	MLLT6	MMP16
MMP2	MMP8	MMP9	MN1	MOS	MPL	MRE11A	MS4A1
MSH2	MSH3	MSH4	MSH6	MSN	MST1R	MTHFR	MTM1
MTMR3	MTOR	MUC1	MUC16	MUC4	MUTYH	MYB	MYBL1
MYBL2	MYC	MYCL1	MYCN	MYD88	MYEOV	MYH1	MYH11
MYH9	MYLK2	MYO1B	MYST4	N4BP2	NBN	NCAM1	NCDN
NCK1	NCK2	NCOA2	NCOA7	NEDD4L	NEIL3	NEK10	NEK11
NEK2	NEK7	NEK8	NEK9	NF1	NF2	NFATC3	NFKB1
NFKB2	NFKBIB	NFKBID	NFKBIE	NFKBIZ	NGFR	NIN	NIPBL
NKD2	NKX2-1	NLE1	NLK	NLRP1	NLRP3	NLRP5	NLRP8
NMUR2	NOS1	NOS2	NOTCH1	NOTCH2	NOTCH3	NOTCH4	NOV
NPM1	NPY2R	NPY5R	NQO1	NR0B1	NRARP	NRAS	NRBP1
NRG2	NRK	NRP2	NTRK1	NTRK2	NTRK3	NUAK1	NUAK2
NUMB	NUMBL	NUP153	NUP214	NUP98	OBSCN	ODZ1	OR8G1
ORAI1	ORAOV1	ORM1	P2RY10	P2RY8	PAG1	PAK1	PAK3
PAK4	PAK6	PAK7	PALB2	PARK2	PARP1	PARP14	PARP2
PARP4	PAX3	PAX5	PAX6	PAX7	PAXIP1	PCDHB2	PCGF2
PCM1	PCNA	PDCD4	PDGFA	PDGFB	PDGFRA	PDGFRB	PDGFRL
PDIA4	PDK1	PDK3	PDPK1	PDZRN4	PEA15	PER1	PERP
PES1	PFKFB1	PFKFB2	PFKFB3	PFKFB4	PGR	PHB	PHF14
PHF20	PHLPP1	PHLPP2	PHOX2B	PIAS1	PIK3C2A	PIK3C2B	PIK3C2G
PIK3C3	PIK3CA	PIK3CB	PIK3CD	PIK3CG	PIK3R1	PIK3R2	PIK3R3
PIM1	PIM2	PKHD1	PKM2	PKMYT1	PKN3	PLA2G4A	PLAG1
PLAGL1	PLAT	PLAU	PLCB1	PLCB4	PLCG1	PLCG2	PLCH1
PLD1	PLD2	PLXNB3	PMAIP1	PML	PMS2	POLE	POLL
POLM	POLN	POLQ	POU1F1	PPARA	PPARG	PPARGC1A	PPM1D
PPM1H	PPM1L	PPP1CC	PPP1R3A	PPP2R1A	PPP2R2B	PPP2R3A	PRDM16
PRDM5	PREX1	PREX2	PRKAA1	PRKAA2	PRKAB1	PRKAB2	PRKACA
PRKACB	PRKACG	PRKAG1	PRKAG2	PRKAG3	PRKAR1A	PRKCA	PRKCD
PRKCE	PRKCG	PRKCH	PRKCI	PRKCZ	PRKD1	PRKD2	PRKDC
PRRC2C	PSEN1	PSEN2	PTCH1	PTCH2	PTEN	PTGFR	PTGS2
PTK2	PTK2B	PTP4A3	PTPN1	PTPN11	PTPN12	PTPN13	PTPN14
PTPN21	PTPN22	PTPN3	PTPN5	PTPN6	PTPN9	PTPRC	PTPRD
PTPRF	PTPRG	PTPRJ	PTPRS	PTPRT	PTPRU	PXN	PYGO2
RAC1	RAD18	RAD21	RAD50	RAD51	RAD51C	RAD54B	RAF1
RALA	RALB	RALGAPA1	RAP1A	RAP1GDS1	RAPGEF2	RAPH1	RARA
RASA1	RASA2	RASA3	RASGRF1	RASGRF2	RASGRP1	RASSF1	RB1
RB1CC1	RBBP4	RBL1	RBL2	RBP2	RBPJ	RECQL4	REG4
REL	RELA	RELB	RET	REV1	REV3L	RFC1	RFC4
RFC5	RFX2	RGL1	RGL2	RHEB	RHOBTB2	RICTOR	RIF1

RING1	*RIPK1*	*RIPK4*	*RNF123*	*RNF213*	*ROBO1*	*ROBO2*	*ROCK1*
ROCK2	*ROR1*	*ROR2*	*ROS1*	*RPL10*	*RPL36A*	*RPS6*	*RPS6KA1*
RPS6KA2	*RPS6KA3*	*RPS6KA4*	*RPS6KA5*	*RPS6KA6*	*RPS6KB1*	*RPS6KB2*	*RPTOR*
RRAS2	*RRM2B*	*RUNX1*	*RUNX1T1*	*RYK*	*SDHB*	*SDHC*	*SDHD*
SENP6	*SERPINB5*	*SESN2*	*SETD2*	*SFN*	*SFRP1*	*SFRP2*	*SGK1*
SGK2	*SGK3*	*SGK494*	*SH3GLB1*	*SHC1*	*SIK1*	*SIRT1*	*SIRT6*
SIX4	*SKP2*	*SLC17A5*	*SLC19A1*	*SLC22A2*	*SLC2A1*	*SLC2A3*	*SLC2A4*
SLC4A4	*SLC6A18*	*SLC6A2*	*SLCO1B3*	*SMAD2*	*SMAD3*	*SMAD4*	*SMAD7*
SMARCA4	*SMARCB1*	*SMC6*	*SMG1*	*SMO*	*SMYD2*	*SNAI1*	*SNAI2*
SNAI3	*SNX13*	*SOCS1*	*SOD2*	*SORL1*	*SOS1*	*SOS2*	*SOX10*
SOX11	*SOX2*	*SPEG*	*SPEN*	*SPO11*	*SPOP*	*SPRED1*	*SPRY1*
SPRY2	*SPTAN1*	*SRC*	*SRF*	*SRPK2*	*SRSF6*	*STAT1*	*STAT3*
STAT4	*STAT5A*	*STAT5B*	*STIL*	*STIM1*	*STK11*	*STK19*	*STK32B*
STK32C	*STK33*	*STK36*	*STMN1*	*STMN3*	*STON2*	*STYK1*	*SUFU*
SULT1A1	*SUZ12*	*SYK*	*SYNE1*	*SYNE2*	*TAB3*	*TACR3*	*TAF15*
TAF1L	*TAL1*	*TANK*	*TBCK*	*TBK1*	*TBKBP1*	*TBX22*	*TCF12*
TCF3	*TCF4*	*TCF7L2*	*TDG*	*TEC*	*TECTA*	*TEK*	*TERT*
TET2	*TEX14*	*TFDP1*	*TFE3*	*TFEB*	*TGFB1*	*TGFB2*	*TGFB3*
TGFBR1	*TGFBR2*	*THBS1*	*THEM4*	*THRAP3*	*TIAM1*	*TICAM1*	*TICAM2*
TK1	*TLE4*	*TLN1*	*TLR2*	*TLR3*	*TLR4*	*TLR7*	*TMEFF2*
TMEM132B	*TMEM161A*	*TMPRSS2*	*TMPRSS6*	*TNF*	*TNFAIP3*	*TNFRSF10A*	*TNFRSF10B*
TNFRSF11A	*TNFRSF1A*	*TNFRSF8*	*TNFSF11*	*TNK2*	*TNKS*	*TNKS2*	*TNNI3K*
TNPO1	*TNPO3*	*TOP1*	*TOP2A*	*TOP2B*	*TOPBP1*	*TP53*	*TP53AIP1*
TP53BP1	*TP63*	*TP73*	*TPD52*	*TPMT*	*TPO*	*TRADD*	*TRAF1*
TRAF2	*TRAF3*	*TRAF4*	*TRAF5*	*TRAF6*	*TRAF7*	*TRIB3*	*TRIM24*
TRIM28	*TRIM33*	*TRIM36*	*TRIM37*	*TRIM47*	*TRIO*	*TRIP11*	*TRRAP*
TSC1	*TSC2*	*TSHR*	*TSPAN31*	*TTBK2*	*TTK*	*TTN*	*TUBD1*
TWF2	*TWIST1*	*TYK2*	*UBA1*	*UBASH3B*	*UBP1*	*UBR4*	*UBR5*
UGT1A1	*UHRF1BP1L*	*ULK1*	*ULK2*	*ULK3*	*UMPS*	*UPP1*	*USP24*
USP28	*USP33*	*USP34*	*USP42*	*USP43*	*USP5*	*USP54*	*USP6NL*
USP7	*USP8*	*USP9X*	*UVRAG*	*VAV1*	*VEGFA*	*VEGFC*	*VEPH1*
VHL	*VPS13B*	*VRTN*	*WEE1*	*WHSC1*	*WIF1*	*WNK1*	*WNK2*
WNK4	*WNT1*	*WNT10B*	*WNT2*	*WNT2B*	*WNT4*	*WNT9B*	*WRN*
WSB1	*WT1*	*WWP2*	*XBP1*	*XIAP*	*XPA*	*XPC*	*XRCC1*
XRCC2	*XRCC3*	*XRCC5*	*XRCC6*	*YWHAQ*	*ZAP70*	*ZBTB16*	*ZC3H12B*
ZEB1	*ZEB2*	*ZMYM2*	*ZMYM4*	*ZNF148*	*ZNF217*	*ZNF384*	*ZNF442*
ZNF831							

Supplementary Table 2. Comparison of Moffitt and TCGA Non-Silent Mutation Rates

Gene	Moffitt Pts	(%)	TCGA Pts	(%)	Pct Diff	Fisher p-value
KRAS	190	(41)	90	(42)	-1	.67
TP53	277	(59)	111	(52)	7	.11
*HLA-A**	84	(18)	4	(2)	16	1.4×10^{-10}
APC	329	(70)	162	(76)	-6	.12
SMAD4	55	(12)	29	(14)	-2	.53
FBXW7	57	(12)	35	(17)	-5	.11
*MUC4**	242	(52)	5	(2)	50	1.1×10^{-43}
BRAF	60	(13)	22	(10)	-3	.45
TCF7L2	45	(10)	30	(14)	-4	.087
PIK3CA	71	(15)	43	(20)	-5	.12
GNAS	45	(10)	23	(11)	-1	.68
TAF1L	56	(12)	18	(8)	4	.19
CSMD3	84	(18)	31	(15)	3	.32
LRP1B	95	(20)	37	(17)	3	.40
*OBSCN**	148	(32)	15	(7)	25	1.1×10^{-13}
*SYNE1**	157	(34)	45	(21)	13	.0011
*TTN**	321	(69)	78	(37)	32	9.5×10^{-15}
*CBX4**	28	(6)	1	(0.5)	6	.0003
*ITGB4**	45	(10)	4	(2)	8	.0002
ADAMTS18	43	(9)	12	(6)	3	.13
FAM123B	46	(10)	24	(11)	-1	.59
*MUC16**	187	(40)	35	(17)	23	4.8×10^{-10}

Note: TCGA data based on 212 samples [2].

* Genes that have substantially lower frequencies (*p*<0.01 in TCGA)

Supplementary Table 3. Demographic features of 10 groups of CRC

Frequencies and percentages of right tumor, stages, MSI, metastasis, and "developing" metastasis in 10 groups

Group	Name	N	Md Age	% Right	No. MSI (%)	Mets (%)	Stage % 1-2	Stage % 3	Stage % 4	Devel. Mets
1a	None	35	65	43	6 (17)	10 (29)	51	26	23	6
1b	B	25	**77** ↑	**84** ↑	**21 (84)** ↑	**3 (12)** ↓	64	24	12	0
2	K	18	67	50	4 (22)	9 (50)	39	22	39	11
3a	P	40	69	41	2 (5)	9 (22)	47	42	**11** ↓	12
3b	BP	20	**75** ↑	60	**10 (50)** ↑	6 (30)	55	15	30	0
4*	A	43	68	37	8 (19)	10 (23)	56	30	14	9
5	KP	18	70	50	2 (11)	8 (44)	33	39	28	17
6	AK	70	66	44	6 (9)	25 (36)	51	23	26	10
7*	AP	113	**60** ↓	**21**↓	**1 (1)** ↓	41 (36)	39	36	25	12
8	AKP	86	65	45	**1 (1)** ↓	**44 (51)** ↑	24	47	29	22
Total		468	66	41	61 (13)	165 (35)	43	33	24	11

Percentages of each of 10 groups and MSI by Primary Location

Location	N	Group^ B/BP	None	K/KP	P	A	AK	AP	AKP	MSI
Cecum	65	8	3	9	8	11	22	**12** ↓	**28** ↑	15
Ascend Rt	100	**25** ↑	8	10	9	8	10	**11** ↓	19	**31** ↑
Transverse	26	12	**19** ↑	8	8	4	27	19	4	**31** ↑
Descend Lt	32	3	3	9	9	6	22	28	19	9
Sigmoid	131	6	6	6	10	9	11	**34** ↑	18	**4** ↓
Rectosig	40	2	2	2	12	8	18	**42** ↑	12	**2** ↓
Rectum	70	**3** ↓	**14** ↑	9	3	14	16	24	17	**6** ↓
Overall	464	10	8	8	8	9	15	24	17	13

Notes:
A = *APC*, K = *KRAS*, P = *TP53*, B = *BRAF.* None = none of the 4 genes are mutated.
* Groups 4 and 7 include 3 and 5 patients with BRAF(*V600E*) mutations, respectively.
^ Groups B and BP, and K and KP were combined together, as their location distrubutions were similar, and their counts were rather low when separated.
AKP muts represents the number of partnering mutations (A, K or P).
There is significantly higher or lower observation than expectation: ↑(↓) for $p<0.05$; ↑↑(↓↓) for $p<0.01$; ↑↑↑(↓↓↓) for $p<0.001$; based on individual chi-square (X^2) contribution from the table cell;
Devel. Mets = Percent of non-stage 4 patients in class who had distant metastasis at tissue collection.

Overall, the median age was 66, 41% had right-sided tumors, 13% were MSI and 35% had distant metastases. The *BRAF* groups (B, BP) (10%) were older, had more right-sided tumors, and higher MSI rates. Only 8 tumors had *BRAF* and *APC* mutations (5 also had *TP53* mutations) and were thus placed into groups 4 and 7; three and one of them were MSI-H, respectively. Group 7 (AP) tumors (24%) were younger, had higher left-sided and MSS rates, while Group 8 (AKP) tumors (18%) were more often MSS and distantly metastatic.

Supplementary Table 4. Association between *KRAS* and *TP53* by *APC* mutation status

A. *APC*mut tumors (N=312)

		*TP53*wt	*TP53*mut	Total
	Group	AK	AKP	
*KRAS*mut	Observed	70	85	155
	Expected	56.1	98.9	
	Group	A	AP	
*KRAS*wt	Observed	43	114	157
	Expected	56.9	100.1	
Total		113	199	312

Pearson r = - 0.18; Fisher p-value = 0.0005

B. *APC*wt tumors (N=156)

		*TP53*wt	*TP53*mut	Total
	Group	K	KP	
*KRAS*mut	Observed	17	18	35
	Expected	17.5	17.5	
	Group	B, Unk	P, BP	
*KRAS*wt	Observed	61	60	121
	Expected	60.5	60.5	
Total		78	78	156

Pearson r = -0.02; Fisher p-value = 0.71

Notes: A = *APC*, K = *KRAS*, P = *TP53*, B = *BRAF*, Unk = tumors with unknown drivers
AKP = mutations in *APC*, *KRAS*, and *TP53;*
The negative association between *KRAS* and *TP53*, however, appears to be induced by the mathematics of the 2×2 table, which requires that the under-representation of *APC*-only tumors be matched in the AKP group.

Supplementary Table 5. *APC* truncation mutation profiles and related other gene mutations

barcode	zone1-2	Zone3-4	Biallelic_loss	msi_high	WNT_ pathway	CTNN	WNT	FAM123B	MACF1	TCF7L2	AXIN1	CDH1	GSK3B	WIF1	TCF4
DS-40207	0	0	0	0	0	0	0	0	0	0	0	0	0	0	0
DS-48717	0	0	0	0	0	0	0	0	0	0	0	0	0	0	0
DS-48788	0	0	0	0	0	0	0	0	0	0	0	0	0	0	0
DS-48888	0	0	0	0	0	0	0	0	0	0	0	0	0	0	0
DS-49422	0	0	0	0	0	0	0	0	0	0	0	0	0	0	0
DS-49449	0	0	0	0	0	0	0	0	0	0	0	0	0	0	0
DS-49454	0	0	0	0	0	0	0	0	0	0	0	0	0	0	0
DS-49513	0	0	0	0	0	0	0	0	0	0	0	0	0	0	0
DS-49664	0	0	0	0	0	0	0	0	0	0	0	0	0	0	0
DS-49843	0	0	0	0	0	0	0	0	0	0	0	0	0	0	0
DS-49844	0	0	0	0	0	0	0	0	0	0	0	0	0	0	0
DS-50544	0	0	0	0	0	0	0	0	0	0	0	0	0	0	0
DS-50587	0	0	0	0	0	0	0	0	0	0	0	0	0	0	0
DS-50607	0	0	0	0	0	0	0	0	0	0	0	0	0	0	0
DS-50712	0	0	0	0	0	0	0	0	0	0	0	0	0	0	0
DS-50740	0	0	0	0	0	0	0	0	0	0	0	0	0	0	0
DS-51020	0	0	0	0	0	0	0	0	0	0	0	0	0	0	0
DS-51043	0	0	0	0	0	0	0	0	0	0	0	0	0	0	0
DS-51610	0	0	0	0	0	0	0	0	0	0	0	0	0	0	0
DS-51652	0	0	0	0	0	0	0	0	0	0	0	0	0	0	0
DS-51809	0	0	0	0	0	0	0	0	0	0	0	0	0	0	0
DS-51948	0	0	0	0	0	0	0	0	0	0	0	0	0	0	0
DS-51995	0	0	0	0	0	0	0	0	0	0	0	0	0	0	0
DS-53059	0	0	0	0	0	0	0	0	0	0	0	0	0	0	0
DS-53114	0	0	0	0	0	0	0	0	0	0	0	0	0	0	0
DS-53116	0	0	0	0	0	0	0	0	0	0	0	0	0	0	0
DS-53211	0	0	0	0	0	0	0	0	0	0	0	0	0	0	0
DS-53230	0	0	0	0	0	0	0	0	0	0	0	0	0	0	0
DS-53303	0	0	0	0	0	0	0	0	0	0	0	0	0	0	0
DS-53317	0	0	0	0	0	0	0	0	0	0	0	0	0	0	0
DS-53324	0	0	0	0	0	0	0	0	0	0	0	0	0	0	0
DS-53359	0	0	0	0	0	0	0	0	0	0	0	0	0	0	0
DS-53460	0	0	0	0	0	0	0	0	0	0	0	0	0	0	0
DS-53489	0	0	0	0	0	0	0	0	0	0	0	0	0	0	0
DS-53528	0	0	0	0	0	0	0	0	0	0	0	0	0	0	0
DS-53679	0	0	0	0	0	0	0	0	0	0	0	0	0	0	0

DS-53750	0	0	0	0	0	0	0	0	0	0	0	0	0	0	0
DS-53783	0	0	0	0	0	0	0	0	0	0	0	0	0	0	0
DS-53800	0	0	0	0	0	0	0	0	0	0	0	0	0	0	0
DS-53957	0	0	0	0	0	0	0	0	0	0	0	0	0	0	0
DS-54043	0	0	0	0	0	0	0	0	0	0	0	0	0	0	0
DS-54051	0	0	0	0	0	0	0	0	0	0	0	0	0	0	0
DS-54054	0	0	0	0	0	0	0	0	0	0	0	0	0	0	0
DS-54730	0	0	0	0	0	0	0	0	0	0	0	0	0	0	0
DS-54783	0	0	0	0	0	0	0	0	0	0	0	0	0	0	0
DS-54833	0	0	0	0	0	0	0	0	0	0	0	0	0	0	0
DS-55343	0	0	0	0	0	0	0	0	0	0	0	0	0	0	0
DS-55398	0	0	0	0	0	0	0	0	0	0	0	0	0	0	0
DS-56069	0	0	0	0	0	0	0	0	0	0	0	0	0	0	0
DS-56330	0	0	0	0	0	0	0	0	0	0	0	0	0	0	0
DS-56334	0	0	0	0	0	0	0	0	0	0	0	0	0	0	0
DS-56551	0	0	0	0	0	0	0	0	0	0	0	0	0	0	0
DS-56604	0	0	0	0	0	0	0	0	0	0	0	0	0	0	0
DS-57046	0	0	0	0	0	0	0	0	0	0	0	0	0	0	0
DS-57048	0	0	0	0	0	0	0	0	0	0	0	0	0	0	0
DS-57055	0	0	0	0	0	0	0	0	0	0	0	0	0	0	0
DS-57433	0	0	0	0	0	0	0	0	0	0	0	0	0	0	0
DS-57616	0	0	0	0	0	0	0	0	0	0	0	0	0	0	0
DS-58510	0	0	0	0	0	0	0	0	0	0	0	0	0	0	0
DS-58532	0	0	0	0	0	0	0	0	0	0	0	0	0	0	0
DS-59213	0	0	0	0	0	0	0	0	0	0	0	0	0	0	0
DS-67098	0	0	0	0	0	0	0	0	0	0	0	0	0	0	0
DS-67931	0	0	0	0	0	0	0	0	0	0	0	0	0	0	0
DS-52005	0	0	0	0	1	0	0	0	0	0	0	1	0	0	0
DS-52925	0	0	0	0	1	0	0	0	0	0	0	1	0	0	0
DS-53117	0	0	0	0	1	0	0	0	0	0	0	1	0	0	0
DS-54038	0	0	0	0	1	0	0	0	0	0	0	1	0	0	0
DS-54189	0	0	0	0	1	0	0	0	0	0	0	1	0	0	0
DS-48894	0	0	0	0	1	0	0	0	0	0	1	0	0	0	0
DS-51972	0	0	0	0	1	0	0	0	0	0	1	0	0	0	0
DS-52367	0	0	0	0	1	0	0	0	0	0	0	0	1	0	0
DS-50597	0	0	0	0	1	0	0	0	1	0	0	0	0	0	0
DS-50631	0	0	0	0	1	0	0	0	1	0	0	0	0	0	0
DS-50925	0	0	0	0	1	0	0	0	1	0	0	0	0	0	0
DS-51754	0	0	0	0	1	0	0	0	1	0	0	0	0	0	0
DS-54223	0	0	0	0	1	0	0	0	1	0	0	0	0	0	0
DS-54289	0	0	0	0	1	0	0	0	1	0	0	0	0	0	0
DS-54405	0	0	0	0	1	0	0	0	1	0	0	0	0	0	0
DS-54718	0	0	0	0	1	0	0	0	1	0	0	0	0	0	0

DS-54926	0	0	0	0	1	0	0	0	1	0	0	0	0	0	0
DS-56142	0	0	0	0	1	0	0	0	1	0	0	0	0	0	0
DS-56635	0	0	0	0	1	0	0	0	1	0	0	0	0	0	0
DS-57052	0	0	0	0	1	0	0	0	1	0	0	0	0	0	0
DS-59550	0	0	0	0	1	0	0	0	1	0	0	0	0	0	0
DS-59556	0	0	0	0	1	0	0	0	1	0	0	0	0	0	0
DS-59974	0	0	0	0	1	0	0	0	1	0	0	0	0	0	0
DS-48937	0	0	0	0	1	0	0	0	0	1	0	0	0	0	0
DS-58843	0	0	0	0	1	0	0	0	0	1	0	0	0	0	0
DS-57472	0	0	0	0	1	0	0	0	0	1	0	1	0	0	0
DS-48055	0	0	0	0	1	0	0	0	0	1	1	0	0	0	0
DS-56979	0	0	0	0	1	0	0	0	1	1	0	0	0	0	1
DS-48957	0	0	0	0	1	0	0	1	0	0	0	0	0	0	0
DS-51877	0	0	0	0	1	0	0	1	0	0	0	0	0	0	0
DS-53682	0	0	0	0	1	0	0	1	0	0	0	0	0	0	0
DS-33635	0	0	0	0	1	0	0	1	1	0	0	0	0	0	0
DS-67984	0	0	0	0	1	0	0	1	1	0	0	0	0	0	0
DS-48893	0	0	0	0	1	0	1	0	0	0	0	0	0	0	0
DS-52126	0	0	0	0	1	0	1	0	0	0	0	0	0	0	0
DS-52847	0	0	0	0	1	0	1	0	0	0	0	0	0	0	0
DS-53053	0	0	0	0	1	0	1	0	0	0	0	0	0	0	0
DS-53168	0	0	0	0	1	0	1	0	0	0	0	0	0	0	0
DS-54564	0	0	0	0	1	0	1	0	0	0	0	0	0	0	0
DS-49315	0	0	0	0	1	0	1	0	1	0	0	0	0	0	0
DS-50620	0	0	0	0	1	0	1	0	1	0	0	0	0	0	0
DS-55411	0	0	0	0	1	0	1	0	1	0	0	0	0	0	0
DS-49208	0	0	0	0	1	1	0	0	0	0	0	0	0	0	0
DS-53516	0	0	0	0	1	1	0	0	0	0	0	0	0	0	0
DS-54031	0	0	0	0	1	1	0	0	0	0	0	0	0	0	0
DS-54362	0	0	0	0	1	1	0	0	0	0	0	0	0	0	0
DS-56796	0	0	0	0	1	1	0	0	0	0	0	0	0	0	0
DS-58478	0	0	0	0	1	1	0	0	0	0	0	0	0	0	0
DS-48639	0	0	0	1	0	0	0	0	0	0	0	0	0	0	0
DS-50754	0	0	0	1	0	0	0	0	0	0	0	0	0	0	0
DS-53382	0	0	0	1	0	0	0	0	0	0	0	0	0	0	0
DS-51155	0	0	0	1	1	0	0	0	0	0	0	1	0	0	0
DS-51783	0	0	0	1	1	0	0	0	0	0	0	1	0	0	0
DS-53730	0	0	0	1	1	0	0	0	0	0	0	0	0	1	0
DS-51711	0	0	0	1	1	0	0	0	0	0	0	1	0	1	0
DS-48913	0	0	0	1	1	0	0	0	1	0	0	0	0	0	0
DS-51827	0	0	0	1	1	0	0	0	1	0	0	0	0	0	0
DS-54750	0	0	0	1	1	0	0	0	1	0	0	0	0	0	0
DS-56351	0	0	0	1	1	0	0	0	1	0	0	0	0	0	0

DS-56820	0	0	0	1	1	0	0	0	1	0	0	0	0	0	0
DS-51215	0	0	0	1	1	0	0	0	1	0	0	1	0	0	0
DS-51893	0	0	0	1	1	0	0	0	1	0	0	0	1	0	0
DS-50760	0	0	0	1	1	0	0	1	1	0	0	0	0	0	0
DS-50817	0	0	0	1	1	0	0	1	1	0	0	0	0	0	0
DS-48658	0	0	0	1	1	0	0	1	1	0	1	0	0	0	1
DS-51942	0	0	0	1	1	0	0	1	0	1	0	1	0	0	0
DS-49669	0	0	0	1	1	0	1	0	0	0	0	0	0	0	0
DS-53687	0	0	0	1	1	0	1	0	0	0	0	0	0	0	0
DS-55390	0	0	0	1	1	0	1	0	0	0	0	0	0	0	0
DS-49665	0	0	0	1	1	0	1	0	0	0	0	0	0	0	1
DS-56326	0	0	0	1	1	0	1	0	0	0	0	1	0	0	0
DS-48712	0	0	0	1	1	0	1	0	1	0	0	0	0	0	0
DS-53232	0	0	0	1	1	0	1	0	1	0	0	0	0	0	0
DS-48909	0	0	0	1	1	0	1	0	1	0	0	1	0	0	0
DS-52929	0	0	0	1	1	0	1	0	1	0	1	0	0	0	0
DS-53945	0	0	0	1	1	0	1	1	1	0	0	0	0	0	0
DS-48645	0	0	0	1	1	1	0	0	0	0	0	0	0	0	0
DS-49373	0	0	0	1	1	1	0	0	0	0	0	0	0	0	0
DS-51584	0	0	0	1	1	1	0	0	0	0	0	0	0	0	0
DS-51798	0	0	0	1	1	1	0	0	0	0	0	0	0	0	0
DS-51982	0	0	0	1	1	1	0	0	0	0	0	0	0	0	0
DS-53731	0	0	0	1	1	1	0	0	0	0	0	1	0	0	0
DS-50258	0	0	0	1	1	1	0	0	1	0	0	0	0	0	0
DS-50698	0	0	0	1	1	1	0	0	1	0	0	0	0	0	0
DS-51671	0	0	0	1	1	1	0	0	1	0	0	0	0	0	0
DS-67027	0	0	0	1	1	1	0	0	1	0	0	0	0	0	0
DS-49496	0	0	0	1	1	1	0	0	1	0	1	0	0	0	0
DS-49521	0	0	0	1	1	1	0	1	0	0	1	0	1	0	0
DS-51886	0	0	0	1	1	1	1	0	0	0	0	0	0	0	0
DS-52095	0	0	0	1	1	1	1	0	0	1	0	0	0	0	0
DS-49127	0	0	0	1	1	1	1	0	1	1	0	0	0	0	0
DS-52864	0	0	0	1	1	1	1	0	1	1	0	0	0	0	0
DS-56888	0	0	0	1	1	1	1	0	1	1	0	0	1	0	0
DS-40203	0	1	0	0	0	0	0	0	0	0	0	0	0	0	0
DS-48595	0	1	0	0	0	0	0	0	0	0	0	0	0	0	0
DS-48682	0	1	0	0	0	0	0	0	0	0	0	0	0	0	0
DS-48795	0	1	0	0	0	0	0	0	0	0	0	0	0	0	0
DS-50692	0	1	0	0	0	0	0	0	0	0	0	0	0	0	0
DS-50851	0	1	0	0	0	0	0	0	0	0	0	0	0	0	0
DS-51018	0	1	0	0	0	0	0	0	0	0	0	0	0	0	0
DS-51803	0	1	0	0	0	0	0	0	0	0	0	0	0	0	0
DS-51941	0	1	0	0	0	0	0	0	0	0	0	0	0	0	0

DS-52121	0	1	0	0	0	0	0	0	0	0	0	0	0	0	0
DS-52681	0	1	0	0	0	0	0	0	0	0	0	0	0	0	0
DS-52756	0	1	0	0	0	0	0	0	0	0	0	0	0	0	0
DS-52771	0	1	0	0	0	0	0	0	0	0	0	0	0	0	0
DS-52790	0	1	0	0	0	0	0	0	0	0	0	0	0	0	0
DS-52838	0	1	0	0	0	0	0	0	0	0	0	0	0	0	0
DS-52882	0	1	0	0	0	0	0	0	0	0	0	0	0	0	0
DS-52940	0	1	0	0	0	0	0	0	0	0	0	0	0	0	0
DS-53052	0	1	0	0	0	0	0	0	0	0	0	0	0	0	0
DS-53156	0	1	0	0	0	0	0	0	0	0	0	0	0	0	0
DS-53453	0	1	0	0	0	0	0	0	0	0	0	0	0	0	0
DS-53505	0	1	0	0	0	0	0	0	0	0	0	0	0	0	0
DS-53835	0	1	0	0	0	0	0	0	0	0	0	0	0	0	0
DS-54072	0	1	0	0	0	0	0	0	0	0	0	0	0	0	0
DS-54148	0	1	0	0	0	0	0	0	0	0	0	0	0	0	0
DS-54195	0	1	0	0	0	0	0	0	0	0	0	0	0	0	0
DS-54327	0	1	0	0	0	0	0	0	0	0	0	0	0	0	0
DS-54393	0	1	0	0	0	0	0	0	0	0	0	0	0	0	0
DS-54429	0	1	0	0	0	0	0	0	0	0	0	0	0	0	0
DS-54452	0	1	0	0	0	0	0	0	0	0	0	0	0	0	0
DS-54556	0	1	0	0	0	0	0	0	0	0	0	0	0	0	0
DS-54569	0	1	0	0	0	0	0	0	0	0	0	0	0	0	0
DS-54679	0	1	0	0	0	0	0	0	0	0	0	0	0	0	0
DS-55324	0	1	0	0	0	0	0	0	0	0	0	0	0	0	0
DS-55383	0	1	0	0	0	0	0	0	0	0	0	0	0	0	0
DS-56094	0	1	0	0	0	0	0	0	0	0	0	0	0	0	0
DS-56803	0	1	0	0	0	0	0	0	0	0	0	0	0	0	0
DS-56894	0	1	0	0	0	0	0	0	0	0	0	0	0	0	0
DS-56962	0	1	0	0	0	0	0	0	0	0	0	0	0	0	0
DS-57013	0	1	0	0	0	0	0	0	0	0	0	0	0	0	0
DS-57035	0	1	0	0	0	0	0	0	0	0	0	0	0	0	0
DS-57927	0	1	0	0	0	0	0	0	0	0	0	0	0	0	0
DS-58258	0	1	0	0	0	0	0	0	0	0	0	0	0	0	0
DS-67827	0	1	0	0	0	0	0	0	0	0	0	0	0	0	0
DS-67949	0	1	0	0	0	0	0	0	0	0	0	0	0	0	0
DS-69689	0	1	0	0	0	0	0	0	0	0	0	0	0	0	0
DS-87910	0	1	0	0	0	0	0	0	0	0	0	0	0	0	0
DS-67980	0	1	0	0	1	0	0	0	0	0	0	0	0	1	0
DS-53132	0	1	0	0	1	0	0	0	0	0	0	0	0	1	1
DS-51050	0	1	0	0	1	0	0	0	1	0	0	0	0	0	0
DS-53298	0	1	0	0	1	0	0	0	1	0	0	0	0	0	0
DS-53482	0	1	0	0	1	0	0	0	1	0	0	0	0	0	0
DS-67030	0	1	0	0	1	0	0	0	1	0	0	0	0	0	0

DS-48833	0	1	0	0	1	0	0	0	0	1	0	0	0	0	0
DS-49498	0	1	0	0	1	0	0	0	0	1	0	0	0	0	0
DS-50536	0	1	0	0	1	0	0	0	0	1	0	0	0	0	0
DS-51766	0	1	0	0	1	0	0	0	0	1	0	0	0	0	0
DS-52569	0	1	0	0	1	0	0	0	0	1	0	0	0	0	0
DS-53728	0	1	0	0	1	0	0	0	0	1	0	0	0	0	0
DS-48670	0	1	0	0	1	0	0	0	1	1	0	0	0	0	0
DS-50521	0	1	0	0	1	0	0	1	0	0	0	0	0	0	0
DS-50626	0	1	0	0	1	0	0	1	0	0	0	0	0	0	0
DS-52871	0	1	0	0	1	0	0	1	0	0	0	0	0	0	0
DS-52888	0	1	0	0	1	0	0	1	0	0	0	0	0	0	0
DS-53129	0	1	0	0	1	0	0	1	0	0	0	0	0	0	0
DS-54674	0	1	0	0	1	0	0	1	0	0	0	0	0	0	0
DS-54751	0	1	0	0	1	0	0	1	0	0	0	0	0	0	0
DS-56668	0	1	0	0	1	0	0	1	1	0	0	0	0	0	0
DS-53095	0	1	0	0	1	0	1	0	0	0	0	0	0	0	0
DS-53143	0	1	0	0	1	0	1	0	0	0	0	0	0	0	0
DS-68849	0	1	0	0	1	0	1	0	0	0	0	0	0	0	0
DS-49826	0	1	0	0	1	0	1	0	1	0	0	0	0	0	0
DS-67953	0	1	0	0	1	0	1	0	1	0	0	0	0	0	0
DS-53295	0	1	0	0	1	0	1	1	0	0	0	0	0	0	0
DS-48649	0	1	0	0	1	1	0	0	0	0	0	0	0	0	0
DS-49425	0	1	0	0	1	1	0	0	0	0	0	0	0	0	0
DS-56858	0	1	0	0	1	1	0	0	0	0	0	0	0	0	0
DS-57955	0	1	0	0	1	1	0	0	0	0	0	0	0	0	0
DS-59932	0	1	0	0	1	1	0	0	0	0	0	0	0	0	0
DS-51875	0	1	0	0	1	1	0	0	0	0	1	0	0	0	0
DS-58341	0	1	0	0	1	1	0	0	1	0	0	0	0	0	0
DS-50648	0	1	0	0	1	1	0	1	0	0	0	0	0	0	0
DS-53972	0	1	0	1	1	0	0	0	1	0	0	0	0	0	0
DS-51086	0	1	0	1	1	1	0	1	0	0	0	0	0	0	1
DS-52912	0	1	0	1	1	1	1	0	0	0	1	0	0	0	1
DS-44878	0	1	1	0	0	0	0	0	0	0	0	0	0	0	0
DS-48592	0	1	1	0	0	0	0	0	0	0	0	0	0	0	0
DS-48921	0	1	1	0	0	0	0	0	0	0	0	0	0	0	0
DS-49637	0	1	1	0	0	0	0	0	0	0	0	0	0	0	0
DS-49730	0	1	1	0	0	0	0	0	0	0	0	0	0	0	0
DS-50529	0	1	1	0	0	0	0	0	0	0	0	0	0	0	0
DS-51087	0	1	1	0	0	0	0	0	0	0	0	0	0	0	0
DS-51527	0	1	1	0	0	0	0	0	0	0	0	0	0	0	0
DS-51878	0	1	1	0	0	0	0	0	0	0	0	0	0	0	0
DS-51923	0	1	1	0	0	0	0	0	0	0	0	0	0	0	0
DS-51977	0	1	1	0	0	0	0	0	0	0	0	0	0	0	0

DS-52111	0	1	1	0	0	0	0	0	0	0	0	0	0	0	0
DS-52151	0	1	1	0	0	0	0	0	0	0	0	0	0	0	0
DS-52359	0	1	1	0	0	0	0	0	0	0	0	0	0	0	0
DS-52889	0	1	1	0	0	0	0	0	0	0	0	0	0	0	0
DS-53661	0	1	1	0	0	0	0	0	0	0	0	0	0	0	0
DS-54062	0	1	1	0	0	0	0	0	0	0	0	0	0	0	0
DS-54129	0	1	1	0	0	0	0	0	0	0	0	0	0	0	0
DS-54255	0	1	1	0	0	0	0	0	0	0	0	0	0	0	0
DS-54355	0	1	1	0	0	0	0	0	0	0	0	0	0	0	0
DS-54724	0	1	1	0	0	0	0	0	0	0	0	0	0	0	0
DS-54737	0	1	1	0	0	0	0	0	0	0	0	0	0	0	0
DS-54845	0	1	1	0	0	0	0	0	0	0	0	0	0	0	0
DS-56118	0	1	1	0	0	0	0	0	0	0	0	0	0	0	0
DS-56176	0	1	1	0	0	0	0	0	0	0	0	0	0	0	0
DS-56348	0	1	1	0	0	0	0	0	0	0	0	0	0	0	0
DS-56384	0	1	1	0	0	0	0	0	0	0	0	0	0	0	0
DS-56964	0	1	1	0	0	0	0	0	0	0	0	0	0	0	0
DS-57008	0	1	1	0	0	0	0	0	0	0	0	0	0	0	0
DS-57026	0	1	1	0	0	0	0	0	0	0	0	0	0	0	0
DS-58141	0	1	1	0	0	0	0	0	0	0	0	0	0	0	0
DS-58440	0	1	1	0	0	0	0	0	0	0	0	0	0	0	0
DS-60341	0	1	1	0	0	0	0	0	0	0	0	0	0	0	0
DS-61202	0	1	1	0	0	0	0	0	0	0	0	0	0	0	0
DS-48808	0	1	1	0	1	0	0	0	1	0	0	0	0	0	0
DS-53115	0	1	1	0	1	0	0	0	1	0	0	0	0	0	0
DS-53121	0	1	1	0	1	0	0	0	1	0	0	0	0	0	0
DS-48655	0	1	1	0	1	0	0	0	0	1	0	0	0	0	0
DS-49417	0	1	1	0	1	0	0	0	0	1	0	0	0	0	0
DS-50772	0	1	1	0	1	0	0	1	0	0	0	0	0	0	0
DS-54439	0	1	1	0	1	0	0	1	0	1	0	0	0	0	0
DS-50723	0	1	1	0	1	0	1	0	0	0	0	0	0	0	0
DS-67162	0	1	1	0	1	0	1	0	0	0	0	0	0	0	0
DS-51121	0	1	1	0	1	0	1	0	1	0	1	0	0	0	0
DS-55407	0	1	1	0	1	0	1	1	0	0	0	0	0	0	0
DS-53969	0	1	1	0	1	1	0	0	0	0	0	0	0	0	0
DS-54782	0	2	0	0	0	0	0	0	0	0	0	0	0	0	0
DS-54928	0	2	0	0	0	0	0	0	0	0	0	0	0	0	0
DS-67065	0	2	0	0	0	0	0	0	0	0	0	0	0	0	0
DS-50931	0	2	0	0	1	0	0	1	0	0	0	0	0	0	0
DS-50573	0	2	0	0	1	0	1	0	0	0	0	0	0	0	0
DS-60296	0	2	0	0	1	1	0	0	0	0	0	0	0	0	0
DS-49796	0	2	1	0	0	0	0	0	0	0	0	0	0	0	0
DS-53494	0	2	1	0	0	0	0	0	0	0	0	0	0	0	0

DS-48823	1	0	0	0	0	0	0	0	0	0	0	0	0	0	0
DS-50568	1	0	0	0	0	0	0	0	0	0	0	0	0	0	0
DS-50796	1	0	0	0	0	0	0	0	0	0	0	0	0	0	0
DS-51630	1	0	0	0	0	0	0	0	0	0	0	0	0	0	0
DS-52022	1	0	0	0	0	0	0	0	0	0	0	0	0	0	0
DS-52131	1	0	0	0	0	0	0	0	0	0	0	0	0	0	0
DS-52369	1	0	0	0	0	0	0	0	0	0	0	0	0	0	0
DS-53385	1	0	0	0	0	0	0	0	0	0	0	0	0	0	0
DS-53481	1	0	0	0	0	0	0	0	0	0	0	0	0	0	0
DS-53736	1	0	0	0	0	0	0	0	0	0	0	0	0	0	0
DS-54009	1	0	0	0	0	0	0	0	0	0	0	0	0	0	0
DS-54089	1	0	0	0	0	0	0	0	0	0	0	0	0	0	0
DS-54391	1	0	0	0	0	0	0	0	0	0	0	0	0	0	0
DS-54697	1	0	0	0	0	0	0	0	0	0	0	0	0	0	0
DS-55379	1	0	0	0	0	0	0	0	0	0	0	0	0	0	0
DS-56101	1	0	0	0	0	0	0	0	0	0	0	0	0	0	0
DS-56310	1	0	0	0	0	0	0	0	0	0	0	0	0	0	0
DS-56626	1	0	0	0	0	0	0	0	0	0	0	0	0	0	0
DS-56683	1	0	0	0	0	0	0	0	0	0	0	0	0	0	0
DS-56842	1	0	0	0	0	0	0	0	0	0	0	0	0	0	0
DS-58234	1	0	0	0	0	0	0	0	0	0	0	0	0	0	0
DS-67013	1	0	0	0	0	0	0	0	0	0	0	0	0	0	0
DS-70294	1	0	0	0	0	0	0	0	0	0	0	0	0	0	0
DS-52210	1	0	0	0	1	0	0	0	0	0	0	1	0	0	0
DS-52853	1	0	0	0	1	0	0	0	0	0	0	0	1	0	0
DS-47369	1	0	0	0	1	0	0	0	1	0	0	0	0	0	0
DS-53246	1	0	0	0	1	0	0	0	1	0	0	0	0	0	0
DS-48764	1	0	0	0	1	0	0	0	0	1	0	0	0	0	0
DS-50827	1	0	0	0	1	0	0	0	0	1	0	0	0	0	0
DS-53272	1	0	0	0	1	0	0	0	0	1	0	0	0	0	0
DS-54041	1	0	0	0	1	0	0	0	0	1	0	0	0	0	0
DS-58275	1	0	0	0	1	0	0	0	0	1	0	0	0	0	0
DS-60252	1	0	0	0	1	0	0	0	0	1	0	0	0	0	0
DS-67994	1	0	0	0	1	0	0	0	0	1	0	0	0	0	0
DS-51676	1	0	0	0	1	0	0	1	0	0	0	0	0	0	0
DS-48607	1	0	0	0	1	1	0	0	0	0	0	0	0	0	0
DS-49325	1	0	0	0	1	1	0	0	0	0	0	0	0	0	0
DS-52942	1	0	0	0	1	1	0	0	0	0	0	0	1	0	0
DS-67955	1	0	0	0	1	1	0	1	1	1	0	0	0	0	0
DS-53424	1	0	0	0	1	1	1	0	0	0	0	0	0	0	0
DS-48929	1	0	0	1	1	0	1	0	1	0	0	0	0	0	0
DS-48789	1	0	0	1	1	1	0	0	0	0	0	0	0	0	0
DS-50855	1	0	0	1	1	1	0	0	0	1	0	0	0	0	0

DS-51220	1	0	0	1	1	1	0	1	0	0	0	0	0	0	0
DS-49718	1	0	1	0	0	0	0	0	0	0	0	0	0	0	0
DS-50725	1	0	1	0	0	0	0	0	0	0	0	0	0	0	0
DS-50739	1	0	1	0	0	0	0	0	0	0	0	0	0	0	0
DS-51821	1	0	1	0	0	0	0	0	0	0	0	0	0	0	0
DS-53191	1	0	1	0	0	0	0	0	0	0	0	0	0	0	0
DS-54015	1	0	1	0	0	0	0	0	0	0	0	0	0	0	0
DS-54589	1	0	1	0	0	0	0	0	0	0	0	0	0	0	0
DS-56678	1	0	1	0	0	0	0	0	0	0	0	0	0	0	0
DS-55384	1	0	1	0	1	0	0	0	1	0	0	0	0	0	0
DS-67956	1	0	1	0	1	0	0	0	1	0	0	0	0	0	0
DS-51932	1	0	1	0	1	0	0	1	0	0	0	0	0	0	0
DS-67844	1	0	1	0	1	0	1	1	1	0	0	1	0	0	0
DS-52320	1	0	1	0	1	1	0	0	0	0	0	0	0	0	0
DS-44604	1	1	0	0	0	0	0	0	0	0	0	0	0	0	0
DS-44863	1	1	0	0	0	0	0	0	0	0	0	0	0	0	0
DS-48739	1	1	0	0	0	0	0	0	0	0	0	0	0	0	0
DS-48827	1	1	0	0	0	0	0	0	0	0	0	0	0	0	0
DS-48857	1	1	0	0	0	0	0	0	0	0	0	0	0	0	0
DS-49395	1	1	0	0	0	0	0	0	0	0	0	0	0	0	0
DS-49435	1	1	0	0	0	0	0	0	0	0	0	0	0	0	0
DS-49441	1	1	0	0	0	0	0	0	0	0	0	0	0	0	0
DS-49459	1	1	0	0	0	0	0	0	0	0	0	0	0	0	0
DS-50652	1	1	0	0	0	0	0	0	0	0	0	0	0	0	0
DS-50685	1	1	0	0	0	0	0	0	0	0	0	0	0	0	0
DS-50687	1	1	0	0	0	0	0	0	0	0	0	0	0	0	0
DS-50788	1	1	0	0	0	0	0	0	0	0	0	0	0	0	0
DS-50812	1	1	0	0	0	0	0	0	0	0	0	0	0	0	0
DS-50890	1	1	0	0	0	0	0	0	0	0	0	0	0	0	0
DS-51088	1	1	0	0	0	0	0	0	0	0	0	0	0	0	0
DS-51769	1	1	0	0	0	0	0	0	0	0	0	0	0	0	0
DS-51797	1	1	0	0	0	0	0	0	0	0	0	0	0	0	0
DS-51997	1	1	0	0	0	0	0	0	0	0	0	0	0	0	0
DS-51998	1	1	0	0	0	0	0	0	0	0	0	0	0	0	0
DS-52837	1	1	0	0	0	0	0	0	0	0	0	0	0	0	0
DS-52992	1	1	0	0	0	0	0	0	0	0	0	0	0	0	0
DS-53055	1	1	0	0	0	0	0	0	0	0	0	0	0	0	0
DS-53138	1	1	0	0	0	0	0	0	0	0	0	0	0	0	0
DS-53139	1	1	0	0	0	0	0	0	0	0	0	0	0	0	0
DS-53189	1	1	0	0	0	0	0	0	0	0	0	0	0	0	0
DS-53408	1	1	0	0	0	0	0	0	0	0	0	0	0	0	0
DS-53644	1	1	0	0	0	0	0	0	0	0	0	0	0	0	0
DS-53664	1	1	0	0	0	0	0	0	0	0	0	0	0	0	0

DS-54307	1	1	0	0	0	0	0	0	0	0	0	0	0	0	0
DS-54363	1	1	0	0	0	0	0	0	0	0	0	0	0	0	0
DS-54503	1	1	0	0	0	0	0	0	0	0	0	0	0	0	0
DS-54841	1	1	0	0	0	0	0	0	0	0	0	0	0	0	0
DS-54925	1	1	0	0	0	0	0	0	0	0	0	0	0	0	0
DS-55355	1	1	0	0	0	0	0	0	0	0	0	0	0	0	0
DS-55388	1	1	0	0	0	0	0	0	0	0	0	0	0	0	0
DS-56080	1	1	0	0	0	0	0	0	0	0	0	0	0	0	0
DS-56293	1	1	0	0	0	0	0	0	0	0	0	0	0	0	0
DS-56397	1	1	0	0	0	0	0	0	0	0	0	0	0	0	0
DS-56421	1	1	0	0	0	0	0	0	0	0	0	0	0	0	0
DS-56829	1	1	0	0	0	0	0	0	0	0	0	0	0	0	0
DS-56830	1	1	0	0	0	0	0	0	0	0	0	0	0	0	0
DS-56949	1	1	0	0	0	0	0	0	0	0	0	0	0	0	0
DS-57041	1	1	0	0	0	0	0	0	0	0	0	0	0	0	0
DS-58548	1	1	0	0	0	0	0	0	0	0	0	0	0	0	0
DS-58694	1	1	0	0	0	0	0	0	0	0	0	0	0	0	0
DS-60313	1	1	0	0	0	0	0	0	0	0	0	0	0	0	0
DS-67914	1	1	0	0	0	0	0	0	0	0	0	0	0	0	0
DS-69122	1	1	0	0	0	0	0	0	0	0	0	0	0	0	0
DS-69164	1	1	0	0	0	0	0	0	0	0	0	0	0	0	0
DS-53249	1	1	0	0	1	0	0	0	0	0	0	1	0	0	0
DS-56740	1	1	0	0	1	0	0	0	0	0	0	1	0	0	0
DS-50805	1	1	0	0	1	0	0	0	0	0	1	1	0	0	0
DS-51028	1	1	0	0	1	0	0	0	1	0	0	0	0	0	0
DS-57686	1	1	0	0	1	0	0	0	1	0	0	0	0	0	0
DS-60353	1	1	0	0	1	0	0	0	1	0	0	0	0	0	0
DS-49685	1	1	0	0	1	0	0	0	0	1	0	0	0	0	0
DS-52122	1	1	0	0	1	0	0	0	0	1	0	0	0	0	0
DS-52232	1	1	0	0	1	0	0	0	0	1	0	0	0	0	0
DS-56376	1	1	0	0	1	0	0	0	0	1	0	0	0	0	0
DS-56608	1	1	0	0	1	0	0	0	0	1	0	0	0	0	0
DS-57829	1	1	0	0	1	0	0	0	0	1	0	0	0	0	0
DS-48778	1	1	0	0	1	0	0	0	1	1	0	0	0	0	0
DS-56309	1	1	0	0	1	0	0	0	1	1	0	0	0	0	0
DS-50248	1	1	0	0	1	0	0	1	0	0	0	0	0	0	0
DS-51528	1	1	0	0	1	0	0	1	0	0	0	0	0	0	0
DS-51679	1	1	0	0	1	0	0	1	0	0	0	0	0	0	0
DS-53146	1	1	0	0	1	0	0	1	0	0	0	0	0	0	0
DS-56302	1	1	0	0	1	0	0	1	0	0	0	0	0	0	0
DS-87902	1	1	0	0	1	0	0	1	0	0	0	0	0	0	0
DS-49375	1	1	0	0	1	0	0	1	1	0	0	0	0	0	0
DS-51680	1	1	0	0	1	0	0	1	1	0	0	0	0	0	0

DS-40199	1	1	0	0	1	0	0	1	0	1	0	0	0	0	0
DS-54552	1	1	0	0	1	0	1	0	0	0	0	0	0	0	0
DS-68634	1	1	0	0	1	0	1	0	0	0	0	0	0	0	0
DS-50283	1	1	0	0	1	0	1	0	1	0	0	0	0	0	0
DS-56660	1	1	0	0	1	0	1	0	0	1	0	0	0	0	0
DS-54437	1	1	0	0	1	0	1	1	0	0	0	0	0	0	0
DS-50613	1	1	0	0	1	1	0	0	0	0	0	0	0	0	0
DS-51984	1	1	0	0	1	1	0	0	0	0	0	0	0	0	0
DS-52979	1	1	0	0	1	1	0	0	0	0	0	0	0	0	0
DS-53487	1	1	0	0	1	1	0	0	0	0	0	0	0	0	0
DS-53710	1	1	0	0	1	1	0	0	0	0	0	0	0	0	0
DS-55364	1	1	0	0	1	1	0	0	0	0	0	0	0	0	0
DS-55377	1	1	0	0	1	1	0	0	0	0	0	0	0	0	0
DS-56140	1	1	0	0	1	1	0	0	0	0	0	0	0	0	0
DS-56963	1	1	0	0	1	1	0	0	0	0	0	0	0	0	0
DS-57006	1	1	0	0	1	1	0	0	0	0	0	0	0	0	0
DS-61205	1	1	0	0	1	1	0	0	0	0	0	0	0	0	0
DS-53198	1	1	0	0	1	1	0	0	1	0	0	0	0	0	0
DS-51531	1	1	0	0	1	1	0	1	0	0	0	0	0	0	0
DS-50720	1	1	0	1	1	0	0	0	1	0	1	0	0	0	1
DS-50877	1	1	0	1	1	0	1	0	0	0	0	0	0	0	0
DS-55003	1	1	0	1	1	1	0	0	1	0	0	0	0	0	0
DS-54758	1	1	0	1	1	1	0	0	1	1	0	0	0	0	0
DS-51156	1	1	0	1	1	1	1	0	0	0	0	0	0	0	0
DS-54160	1	1	0	1	1	1	1	1	0	0	0	0	0	0	0
DS-48725	1	1	1	0	0	0	0	0	0	0	0	0	0	0	0
DS-49116	1	1	1	0	0	0	0	0	0	0	0	0	0	0	0
DS-49639	1	1	1	0	0	0	0	0	0	0	0	0	0	0	0
DS-49716	1	1	1	0	0	0	0	0	0	0	0	0	0	0	0
DS-50896	1	1	1	0	0	0	0	0	0	0	0	0	0	0	0
DS-56647	1	1	1	0	0	0	0	0	0	0	0	0	0	0	0
DS-68891	1	1	1	0	0	0	0	0	0	0	0	0	0	0	0
DS-51202	1	1	1	0	1	0	1	0	0	1	0	0	0	0	0
DS-54013	1	2	0	0	0	0	0	0	0	0	0	0	0	0	0
DS-49695	1	3	0	0	1	0	0	0	1	1	0	1	0	1	1
DS-56867	2	0	0	0	0	0	0	0	0	0	0	0	0	0	0
DS-56998	2	0	0	0	0	0	0	0	0	0	0	0	0	0	0
DS-57060	2	0	0	0	0	0	0	0	0	0	0	0	0	0	0
DS-51918	2	0	0	0	1	0	0	0	1	0	0	0	0	0	0
DS-53725	2	0	0	0	1	0	0	0	0	1	0	0	0	0	0
DS-52815	2	0	0	1	1	0	0	0	1	0	0	0	0	0	0
DS-51952	2	0	0	1	1	1	1	1	1	1	0	0	1	1	0
DS-49699	2	1	0	0	1	1	0	0	0	0	0	0	0	0	0

DS-50776	2	1	0	0	1	1	1	1	1	0	0	0	1	1	1
DS-59919	2	1	0	1	1	1	1	1	0	1	1	0	0	0	0

Note that 0 = “No” and 1 = “Yes”.

Supplementary Table 6. Counts of *APC* truncating mutations by sample using the TCGA COAD data.

**No. of *APC* truncating mutations**	**0**	**1**	**2**	**3**
***APC* copy number change**	0	0	0	0
**No. of samples**	46	74	38	3

**No. of *APC* truncating mutations**	**0**	**1**	**2**
***APC* copy number change**	-1	-1	-1
**No. of samples**	6	32	3

**No. of *APC* truncating mutations**	**0**	**2**	**0**
***APC* copy number change**	+1	+1	-2
**No. of samples**	1	5	1

Notes: 209 total samples with somatic mutation, CAN, and expression data; 60.3% of total have 0 or 1 hits. Data downloaded from: https://tcga-data.nci.nih.gov/docs/publications/coadread_2012/

Supplementary Table 7. Immunohistochemical staining (Allred scores) of β-catenin in 52 CRCs.

Barcode	Allred scores (nuclear)	Allred scores (cyto)	Allred scores (membranous)
DS-33635	4	7	7
DS-48055	0	7	7
DS-48893	3	8	8
Ds-49315	5	8	7
DS-50925	0	7	8
DS-51043	8	8	0
DS-51652	0	6	8
DS-51982	0	7	8
DS-53114	5	8	8
DS-53211	0	7	8
DS-54051	7	8	8
DS-54564	7	7	4
DS-54783	7	7	5
DS-56326	0	6	7
DS-47369	7	8	8
DS-48607	7	8	7
DS-48764	8	8	6
DS-49826	5	7	7
DS-51803	5	8	7
DS-51941	0	5	8
DS-52210	5	7	8
DS-52681	7	6	6
DS-52790	6	7	7
DS-52838	6	6	8
DS-52853	5	7	8
DS-53453	5	7	7
DS-54041	7	8	5
DS-58258	7	6	7
DS-67955	7	7	5
DS-67980	7	6	7
DS-70294	6	7	7
DS-40199	4	7	8
DS-44878	5	8	7
DS-48857	7	8	5
DS-49796	0	7	8
DS-50687	3	6	7
DS-51797	6	7	7
DS-51977	7	7	6

DS-51997	6	7	7
DS-52151	4	7	7
DS-52320	7	8	4
DS-53191	6	5	7
DS-54129	6	7	7
DS-54363	7	6	6
DS-54589	5	7	7
DS-56376	5	7	7
DS-56963	0	7	8
DS-58341	4	6	7
DS-60296	7	6	6
DS-60353	0	7	8
DS-49639	5	8	7
Ds-56293	2	7	8

Supplemental Table 8. Five-class classification and CMS classification (N=458)

	Classes	N	%CMS1	%CMS2	%CMS3	%CMS4	%NA	%Total
**0**	"*APC*wt"	151	34 ↑↑↑	8 ↓↓↓	12	28	18	100
**1**	"A(1), A(1)K, A(1)P"	138	6 ↓↓	36 ↑	13	24	21	100
**2**	"A(2), A(2)K, A(2)P"	83	13	35	18	17	17	100
**3**	"APC(1)KP"	45	9	33	11	27	20	100
**4**	"APC(2)KP"	41	5	24	20	27	24	100

Note:
1) A−*APC*, K−*KRAS,* P−*TP53*
Class 0: *APC* wild type**; Class 1:** *APC(1), APC(1)*/*KRAS*, *APC(1)*/*TP53*; **Class 2:** *APC(2), APC(2)*/*KRAS*, *APC(2)*/*TP53*; **Class 3:** *APC(1)/KRAS*/*TP53*; **Class 4**: *APC(2)/KRAS*/*TP53.* While A(1) or *APC* (1) represents one *APC* mutation, A(2) or *APC* (2) represents two *APC* mutations.
2)↑(↓) for p<0.05; ↑↑(↓↓) for p<0.01; ↑↑↑(↓↓↓) for p<0.001; based on individual chi-square contribution from the table cell.

Supplementary Table 9. Cox model predictors of overall survival of MSS tumors (N=399) (Models 1-4)

	Model 1		Model 2	
Covariable	HR	p-value	HR	p-value
APC			0.47	0.0008
BRAF			2.23	0.012
**2 *APC* muts**			1.63	0.019
**AKP**			1.45	0.08
**Metastasis**	4.44	<.0001	4.54	<.0001
**AGE(≥70)**	1.10	<.0001	1.09	<.0001
**LR χ2**	75.7	<.0001	98.8	<.0001

	Model 3		Model 4		Model 5	
Covariable	HR	p-value	HR	p-value	HR	p-value
APC			0.52	0.003	0.62	0.015
BRAF			1.55	0.23	1.50	0.26
**2 *APC* muts**			1.57	0.034		
**AKP**			1.37	0.14		
***APC*(2)KP**					2.03	0.007
**CMS1**	3.48	<.0001	2.39	0.012	2.55	0.007
**CMS3**	1.76	0.06	1.45	0.22	1.53	0.16
**CMS4**	1.34	0.22	1.24	0.37	1.24	0.37
**CMS_NA**	1.82	0.02	1.53	0.10	1.51	0.11
**Metastasis**	4.99	<.0001	4.86	<.0001	4.82	<.0001
**AGE(≥70)**	1.09	<.0001	1.09	<.0001	1.09	<.0001
**LR χ2**	92.6	<.0001	105.4	<.0001	105.1	<.0001

Supplementary References

1 Samowitz, W. S. *et al.* APC mutations and other genetic and epigenetic changes in colon cancer. *Molecular cancer research : MCR* **5**, 165-170, doi:10.1158/1541-7786.MCR-06-0398 (2007).

2 Cancer Genome Atlas, N. Comprehensive molecular characterization of human colon and rectal cancer. *Nature* **487**, 330-337, doi:10.1038/nature11252 (2012).

3 Herbst, A. *et al.* Comprehensive analysis of beta-catenin target genes in colorectal carcinoma cell lines with deregulated Wnt/beta-catenin signaling. *BMC genomics* **15**, 74, doi:10.1186/1471-2164-15-74 (2014).

4 Dry, J. R. *et al.* Transcriptional pathway signatures predict MEK addiction and response to selumetinib (AZD6244). *Cancer research* **70**, 2264-2273, doi:10.1158/0008-5472.CAN-09-1577 (2010).

5 Loboda, A. *et al.* EMT is the dominant program in human colon cancer. *BMC Med Genomics* **4**, 9, doi:10.1186/1755-8794-4-9 (2011).
